# JAK2 Inhibition Augments the Anti-Proliferation Effects by AKT and MEK Inhibition in Triple-Negative Breast Cancer Cells

**DOI:** 10.3390/ijms26136139

**Published:** 2025-06-26

**Authors:** Kyu Sic You, Tae-Sung Kim, Su Min Back, Jeong-Soo Park, Kangdong Liu, Yeon-Sun Seong, Dong Joon Kim, Yong Weon Yi

**Affiliations:** 1 Department of Biochemistry, College of Medicine, Dankook University, Cheonan-si 31116, Republic of Korea; 2 Multidrug-Resistant Refractory Cancer Convergence Research Center (MRCRC), Dankook University, Cheonan-si 31116, Republic of Korea; 3 Department of Pathophysiology, School of Basic Medical Sciences, Academy of Medical Science, College of Medicine, Zhengzhou University, Zhengzhou 450008, China; 4 China-US (Henan) Hormel Cancer Institute, Zhengzhou 450008, China; 5 Department of Microbiology, College of Medicine, Dankook University, Cheonan-si 31116, Republic of Korea

**Keywords:** anticancer, combination, JAK2/STAT3 pathway, MEK/ERK pathway, PI3K/AKT pathway, protein kinase inhibitors, triple-negative breast cancer

## Abstract

Janus kinase 2 (JAK2) inhibitors have gained regulatory approval for treating various human diseases. While the JAK2/signal tranducer and activator of transcription 3 (STAT3) pathway plays a role in tumorigenesis, JAK2/STAT3 inhibitors have shown limited therapeutic efficacy in triple-negative breast cancer (TNBC). In this study, we assessed the antiproliferative effects of clinically approved JAK2 inhibitors in TNBC cell lines (MDA-MB-231 and HS578T) using the MTT assay. Among the four JAK2 inhibitors evaluated (fedratinib, cerdulatinib, peficitinib, and filgotinib), fedratinib significantly inhibited the proliferation of TNBC cells with IC_50_ values below 2 μM. Fedratinib also demonstrated superior efficacy in inhibiting long-term colony formation compared to other JAK2 inhibitors. Western blot analyses showed that fedratinib uniquely inhibits the phosphoinositide 3-kinase (PI3K)/AKT pathway and moderately affects the MAP kinase/ERK kinase (MEK)/extracellular signal-regulated kinase (ERK) pathway, in addition to targeting JAK2/STAT3 signaling. Moreover, fedratinib distinctly decreased MYC and cyclin D1 protein levels while inducing poly (ADP-ribose) polymerase (PARP) cleavage and apoptotic cell death more effectively than other JAK2 inhibitors. We next investigated the effects of simultaneously inhibiting JAK2/STAT3 together with the MEK/ERK or PI3K/AKT pathways, as well as the impact of triple pathway inhibition. Notably, combining ceduratinib with either cobimetinib (MEK inhibitor) and ipatasertib (AKT inhibitor) or trametinib (MEK inhibitor) and alpelisib (PI3K inhibitor) mimicked the effects of fedratinib on the cell proliferation, MYC and cyclin D1 suppression, and pro-apoptotic protein induction. These finding suggest that JAK2 inhibition enhances the anticancer effects of concurrent MEK/ERK and PI3K/AKT pathway inhibition, while JAK2 inhibition alone shows minimal efficacy in TNBC cells.

## 1. Introduction

TNBC is the most aggressive type of breast cancer, characterized by the absence of estrogen receptor (ER) and progesterone receptor (PR) expression, and lack of human epidermal growth factor receptor 2 (HER2) amplification [1,2,3,4]. Representing 15–20% of breast cancer cases, TNBC exhibits intrinsic resistant against targeted therapeutics for epidermal growth factor receptor (EGFR) and MET, despite their frequent amplification in TNBC [1,2,4,5,6,7,8,9,10,11,12]. Currently, five targeted therapies have received the US Food and Drug Administration (FDA) approval: two poly [adenosine diphosphate (ADP)-ribose] polymerase 1 (PARP1) inhibitors (olaparib and talazoparib); a programmed cell death ligand 1 (PD-L1) inhibitor atezolizumab, an antibody targeting programmed cell death 1 (PD1) pembrolizumab, and an antibody drug conjugate (ADC) sacituzumab govitecan [3,13]. However, due to limited clinical responses, alternative therapeutic strategies remain crucial for effective management of TNBC [3,13].

STAT3 functions as a transcriptional regulator responding to various cytokines [14]. The activation of STAT3 occurs through phosphorylation at 705 tyrosine residue (Y705), primarily mediated by JAK2, one of four JAK kinase family members [JAK1, JAK2, JAK3, and tyrosine kinase 2 (TYK2)] [15]. The JAK2/STAT3 pathway plays significant roles in tumorigenesis and tumor progression across multiple cancer types [16,17,18,19,20]. Currently, eleven small-molecular JAK inhibitors (JAKis) have received global regulatory approval for various conditions, including myelofibrosis, graft-versus-host disease (GvHD), polycythemia vera, alopecia areata, rheumatoid arthritis, psoriatic dermatitis, atopic dermatitis, and ulcerative colitis [21,22]. Although the JAK2/STAT3 pathway has been suggested as a potential therapeutic target for treatment of TNBC, clinical application of JAK2/STAT3 inhibitors has not been fully successful [23]. Our previous research demonstrated that the inhibition of JAK1/2 by CP690550 [24] is not sufficient to inhibit the proliferation of TNBC cells [25]. Rather, the inhibition of acetylation of STAT3 at lysine 685 is necessary for anticancer effects of a small molecule inhibitor SH-I-14 though disruption of STAT3-DNA (cytosine-5)-methyltransferase 1 (DNMT1) interaction, leading to the induction of re-expression of tumor suppressor gene products such as von Hippel-Lindau disease tumor suppressor (VHL) and PDZ and LIM domain protein 4 (PDLIM4) [23].

Here, we report the contribution of JAK2 inhibitor (JAK2i) to augment the MEK/ ERK and PI3K/AKT pathway inhibition in TNBC cells. We found that the inhibition of the JAK2/STAT3 pathway alone is not sufficient to reduce the proliferation of TNBC cells, whereas JAK2i is required for the enhancement of anticancer effects by co-inhibition of MEK and AKT or co-inhibition of MEK and PI3K via downregulating levels of MYC and cyclin D1 proteins and inducing apoptosis in TNBC cells. Our data suggest that the inhibition of the JAK2/STAT3 pathway provides additional therapeutic benefit for the combination of MEK/ERK and PI3K/AKT inhibition.

## 2. Results

### 2.1. Fedratinib Inhibits the Proliferation of TNBC Cells

**Figure 1:**
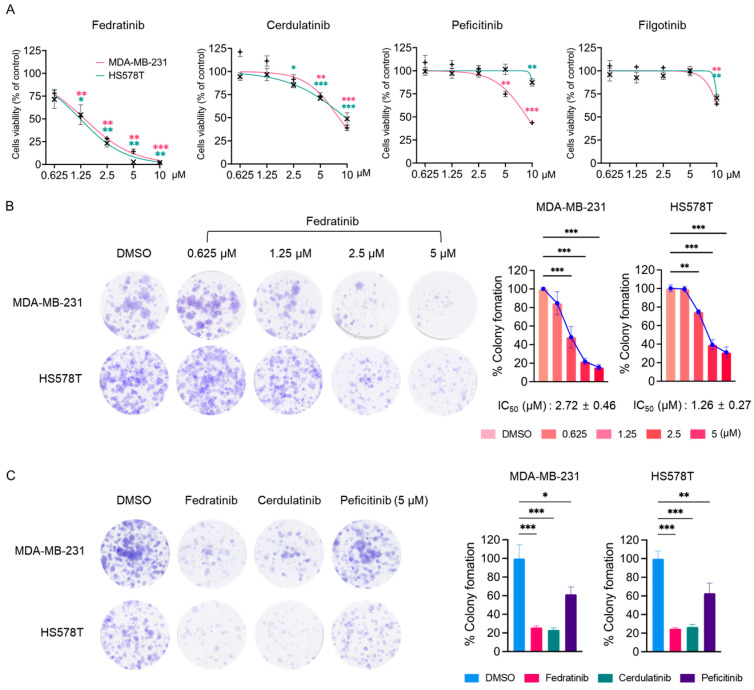
JAK2is reduces the viability and colony formation in TNBC cells. (**A**) Cell viability of MDA-MB-231 and HS578T cells following 72 h treatment with indicated JAK2is, assessed by MTT assay. (**B**) Colony formation of MDA-MB-231 and HS578T cells (1 × 10^3^ cells/well) treated with fedratinib at indicated concentration for 24 h, followed by 10–14 days of growth in inhibitor-free medium. Representative images and quantification of crystal violet-stained colonies are shown. (**C**) Clonogenic survival of TNBC cells treated with fedratinib, cerdulatinib, or peficitinib (5 μM) for 24 h. followed by growth in inhibitor-free medium. Data present means ± SEMs from three independent experiments. * *p* < 0.05, ** *p* < 0.01, and *** *p* < 0.005.

During the course of screening of the US FDA-approved protein kinase inhibitor (PKI) library using MTT assay in TNBC cells [4,5,6,26,27,28,29,30], we observed notable effects of JAK2is. In agreement with our previous findings [25], three JAK2is (cerdulatinib, peficitinib, and filgotinib) showed minimal effects on the proliferation of two TNBC cell lines (Table 1 and Figure 1A). However, fedratinib distinctly reduced the proliferation of MDA-MB-231 and HS578T cells with IC_50_ values of 1.38 ± 0.06 and 1.23 ± 0.19 μM, respectively (Table 1 and Figure 1A). While all these small molecules target JAK2 [31,32,33,34,35], fedratinib specifically inhibits JAK2 with limited activity against other JAK family members [31]. Additionally, fedratinib targets FMS-like tyrosine kinase 3 (FLT3) and rearranged during transfection (RET), albeit with lower potency than its JAK2 inhibition [31]. In contrast, cerdulatinib was developed as a dual inhibitor of spleen tyrosine kinase (SYK) and JAKs, with IC_50_ values below 40 nM [32]. Filgotinib demonstrates potent inhibitory activity against both JAK1 and JAK2 [33], while peficitinib inhibits all JAK family members in nanomolar ranges [34,35]. As demonstrated in Table 1 and Figure 1A, fedratinib uniquely suppressed the proliferation of HS578T and MDA-MB-231 cells with IC_50_ values below 2 μM, whereas the other inhibitors showed only marginal effects with IC_50_ values exceeding 8 μM. In colony formation assay, fedratinib effectively reduced the long-term survival of both TNBC cell lines, with IC_50_ values of 2.72 ± 0.46 μM (MDA-MB-231) and 1.26 ± 0.27 μM (HS578T) (Figure 1B). Notably, at 5 μM concentration, cerdulatinib showed comparable effects to fedratinib on long-term survival, while peficitinib did not (Figure 1C).

### 2.2. Fedratinib Inhibits the PI3K/AKT Pathway and Marginally Modulates the MEK/ERK Pathway in TNBC Cells

**Figure 2:**
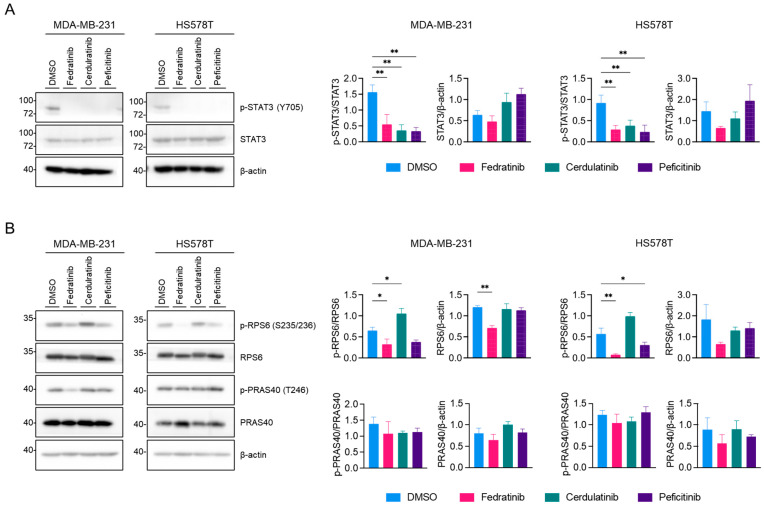
Fedratinib inhibits JAK2/STAT3 and PI3K/AKT signaling in TNBC cells. (**A**,**B**) Western blot analysis of signaling proteins in TNBC cells treated with fedratinib, cerdulatinib, or peficitinib (5 μM, 24 h). Representative images from three independent experiments were shown with densitometric quantification (mean ± SEM). * *p* < 0.05 and ** *p* < 0.01.

We further characterized three JAK2is—fedratinib, cerdulatinib, and peficitinib—using western blot analyses. As expected, all these JAK2is reduced the levels of phosphorylation of STAT3 at tyrosine 705 (Y705), a modification catalyzed by JAK2 [36], in TNBC cell lines (Figure 2A; Original WB images are available as Supplementary Materials, File S1: Original WB images). Our previous research demonstrated that the inhibition of phospho (p)-STAT3 (Y705) alone is insufficient to reduce the proliferation of TNBC cells [25]. Given that fedratinib also targets two receptor tyrosine kinases (RTKs), FLT3 and RET (Table 1), we examined two major downstream pathways of RTK, the PI3K/AKT and MEK/ ERK pathways, in TNBC cell lines. As shown in Figure 2B, fedratinib decreased the levels of p-ribosomal protein S6 (p-RPS6) (S235/236), a downstream target of AKT [37], in both HS578T and MDA-MB-231 cells. While peficitinib also reduced the level of p-RPS6 (S235/236), this reduction did not reach statistical significance in MDA-MB-231 cells. In contrast, cerdulatinib increased p-RPS6 (S235/236) levels in both cell lines. Interestingly, phosphorylation of proline-rich AKT1 substrate 40 (PRAS40) at T248, a direct substrate of AKT [38], remained unchanged by JAK2is. This maintenance of p-PRAS40 (T246) levels might result from compensatory phosphorylation by other kinases, particularly PIM1 [39], which has emerged as a promising therapeutic target for TNBC treatment [40,41,42,43].

**Figure 3:**
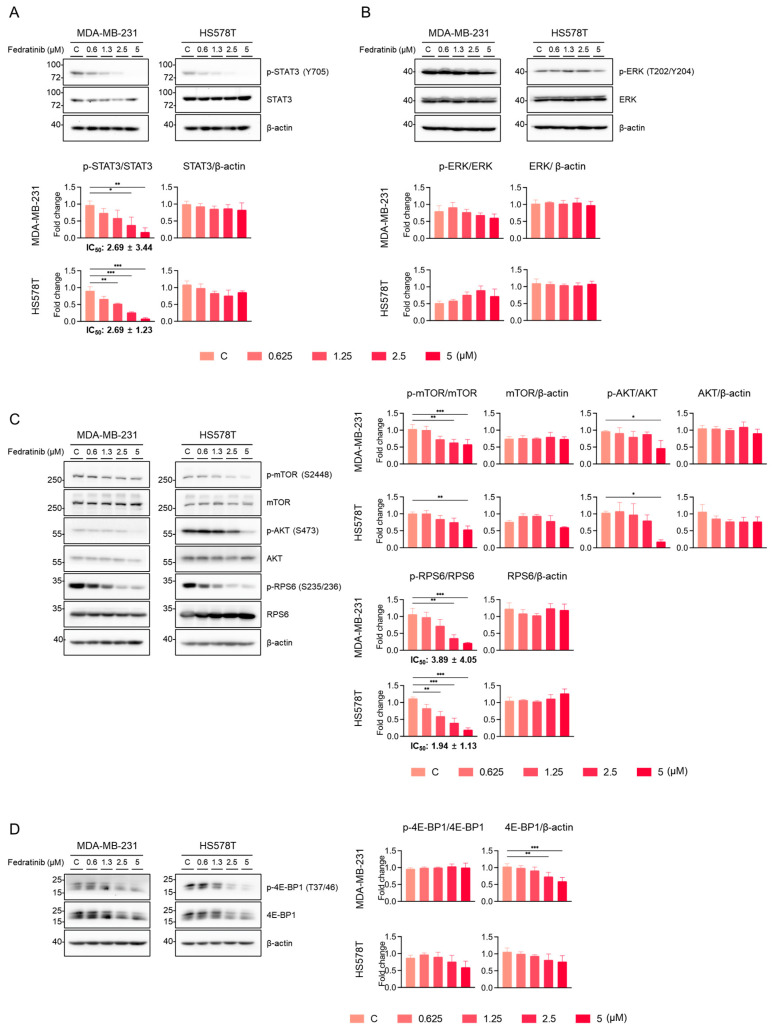
Fedratinib inhibits multiple signaling pathways in TNBC cells. MDA-MB-231 and HS578T cells were treated with increasing concentrations of fedratinib for 24 h. (**A**) STAT3 phosphorylation (Y705), (**B**) ERK phosphorylation (T202/Y204), (**C**) PI3K/AKT pathway proteins [p-mTOR (S2448), p-AKT (S473), and p-RPS6 (S235/236)], and (**D**) 4E-BP1 levels were analyzed by Western blot, with β-actin as loading control. Representative blot images from three independent experiments are shown with densitometric quantification (mean ± SEM). * *p* < 0.05, ** *p* < 0.01, and *** *p* < 0.005.

We further evaluated the effects of fedratinib on key signaling pathways. As anticipated, fedratinib dose-dependently reduced the levels of p-STAT3 (T705), with IC_50_ values of 2.69 ± 3.44 μM for MDA-MB-231 and 2.69 ± 1.23 μM for HS578T cells (Figure 3A). Under these experimental conditions, fedratinib showed differential effects on p-ERK1/2 (T202/Y204), the sole known substrate for MEK1/2 [44]: modest dose-dependent reduction in MDA-MB-231 cells and a slight, statistically insignificance increase in HS578T cells (Figure 3B).

The inhibitory effects of fedratinib on the levels of p-mammalian target of rapamycin (p-mTOR) (S2448), p-AKT (S473), and p-RPS6 (S235/236) became apparent at 2.5 or 5 μM in TNBC cells (Figure 3C). Since S2448 of mTOR is a substrate for AKT1 [also known as protein kinase B (PKB)] [45], the reduction in p-mTOR (S2448) indicates that fedratinib inhibits AKT activity, likely through indirect inhibition of upstream RTKs such as FLT3 or RET. Moreover, as mTOR complex 2 (mTORC2) mediates AKT S473 phosphorylation for full activation [46], the decreased p-AKT (S473) levels further confirm the inhibition of the PI3K/AKT pathway by fedratinib. The levels of p-RPS6 (S235/236) were dose-dependently reduced by fedratinib with IC_50_ values of 3.89 ± 4.05 μM for MDA-MB-231 and 1.94 ± 1.33 μM for HS578T cells (Figure 3C). eIF4E-binding protein 1 (4E-BP1), an oncogenic convergent target integrating the MEK/ERK and PI3K/AKT pathways [47], showed reduced phosphorylation at T37/46 upon fedratinib treatment in a dose-dependent manner (Figure 3D). Notably, densitometric analyses revealed a significant reduction in the total 4E-BP1 levels in MDA-MB-231 cells and a marginal reduction in HS578T cells, previously unreported drug effect in cancer cells.

### 2.3. Fedratinib Inhibited the MEK/ERK Pathway in TNBC Cells in a Time- and Cell Line-Dependent Manners

**Figure 4:**
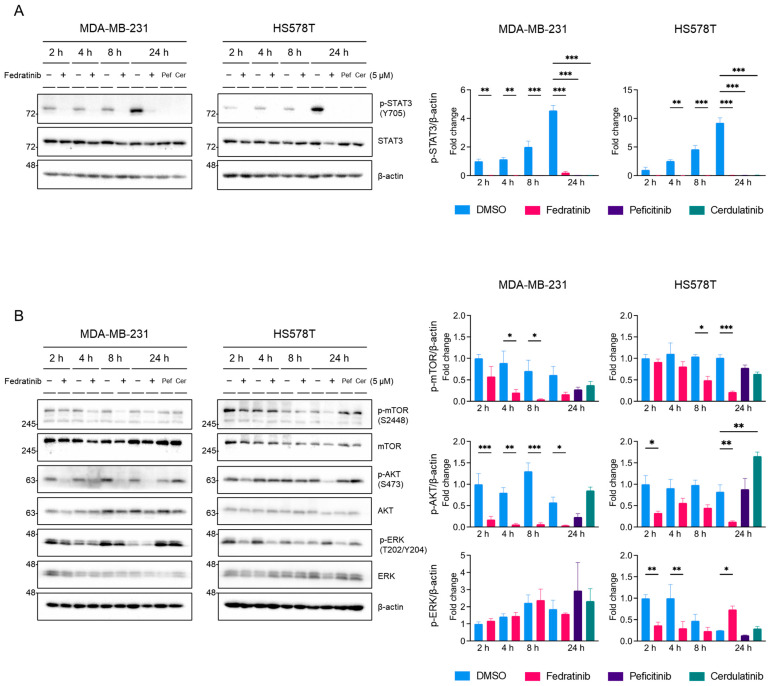
Time-dependent inhibition of signaling pathways by fedratinib in TNBC cells. MDA-MB-231 and HS578T were treated with DMSO or fedratinib (5 μM) for indicated times, or cerdulatinib (5 μM) and peficitinib (5 μM) 24 h. (**A**) STAT3 phosphorylation (Y705) and (**B**) phosphorylation status of PI3K/AKT and MEK/ERK pathway proteins were analyzed by Western blot, with β-actin as a loading control. Data present mean ± SEM from three independent experiments. * *p* < 0.05, ** *p* < 0.01, and *** *p* < 0.005.

To clarify the effects of fedratinib on the MEK/ERK pathway, we conducted time-course analyses in TNBC cells. Treatment with 5 μM fedratinib reduced p-STAT3 (Y705) levels to near undetectable levels as early as 2 h post-treatment, maintaining suppression for 24 h, while the control (DMSO-treated) cells showed increasing p-STAT3 (Y705) levels over time (Figure 4A). Similarly, 5 μM peficitinib and cerdulatinib suppressed p-STAT3 (Y705) levels at 24 h post-treatment (Figure 4A). The effects on p-mTOR (S2448) and p-AKT (S473) showed cell line specificity (Figure 4B): In MDA-MB-231 cells, the levels of p-mTOR (S2448) were decreased by fedratinib significantly at as early as 4 h post-treatment, though sustained suppression lacked statistical significance. In HS578T cells, a significant decrease in p-mTOR (S2448) was observed at 8 h post-treatment and persisted through 24 h. p-ATK (S473) inhibition paralleled p-STAT3 (Y705) patterns in MDA-MB-231 cells, showing sustained suppression from 2 h onward, while HS578T cells exhibited significant suppression only at 2 and 24 h post-treatment.

Peficitinib and cerdulatinib showed inconsistent effects between cell lines: peficitinib reduced p-mTOR (S2448) and p-AKT (S473) levels in MDA-MB-231 cells without statistical significance, while cerdulatinib showed minimal effect on p-mTOR (S2448) levels in both cell lines and increased p-AKT (S473) levels in HS58T cells (Figure 4B). Fedratinib weakly decreased p-ERK (T202/Y204) levels at 24 h post-treatment in MDA-MB-231 (not statistically significant) and at 2 and 4 h post-treatment in HS578T cells (Figure 4B), but markedly increased the levels of p-ERK (T202/Y204) at 24 h in HS578T cells. Peficitinib and cerdulatinib showed minimal effects on ERK phosphorylation. These findings indicate that fedratinib consistently inhibits the PI3K/AKT pathway, while affecting the MEK/ERK pathway in time- and cell line- dependent manners [48,49,50]. Notably, p-ERK (T202/Y204) levels fluctuated in the control cells, as previously reported in normal mammary epithelial cells [51], which may influence TNBC proliferation.

**Figure 5:**
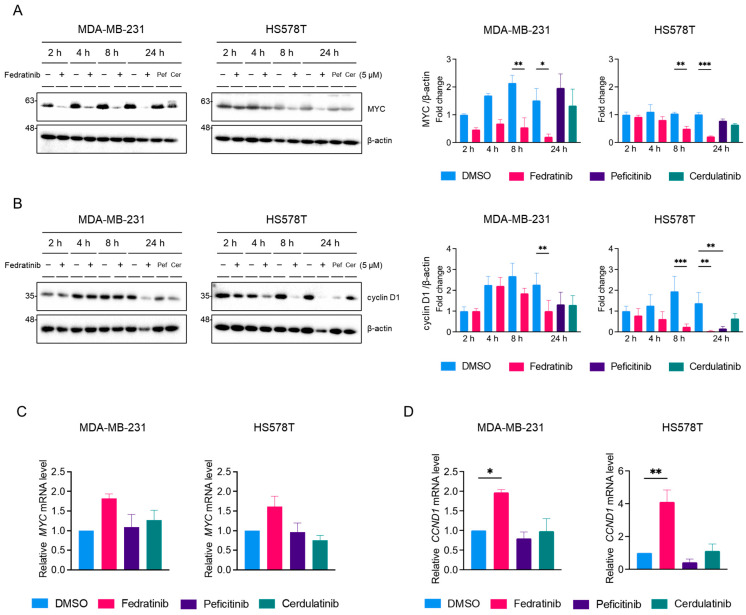
Fedratinib downregulates MYC and cyclin D1 expressions in TNBC cells. MDA-MB-231 and HS578T were treated with fedratinib (5 μM) for indicated times, or peficitinib and cerdulatinib (5 μM) for 24 h. Western blot analysis of (**A**) MYC and (**B**) cyclin D1 (**B**) protein levels, with β-actin as a loading control. (**C**) Relative *MYC* and (**D**) *CCND1* mRNA levels measured by quantitative RT-PCR. Data represent mean ± SEM from three independent experiments. * *p* < 0.05, ** *p* < 0.01, and *** *p* < 0.005.

Proto-oncogene MYC and cyclin D1 are common downstream targets of the JAK2/STAT3, PI3K/AKT, and MEK/ERK pathways [48,49,50]. Western blot analyses revealed that fedratinib, but not peficitinib or cerdulatinib, suppressed MYC protein levels in both TNBC cell lines (Figure 5A). This reduction of MYC protein levels became apparent by 8 h and persisted through 24 h post-treatment in both TNBC cell lines. Similarly, fedratinib decreased cyclin D1 levels in both cell lines (Figure 5B), while peficitinib-induced downregulation of cyclin D1 was observed only in HS578T cells.

To determine whether these protein level changes resulted from transcriptional regulation, we performed quantitative real-time PCR (RT-PCR) analyses. Interestingly, fedratinib treatment increased *MYC* mRNA levels in both cell lines (Figure 5D). Similarly, *CCND1* (the gene encoding cyclin D1) mRNA levels were also elevated by fedratinib in both cell lines (Figure 5D). Treatment with peficitinib or cerdulatinib showed minimal significant effects.

### 2.4. Fedratinib Induces Apoptotic Cell Death in TNBC Cells

**Figure 6:**
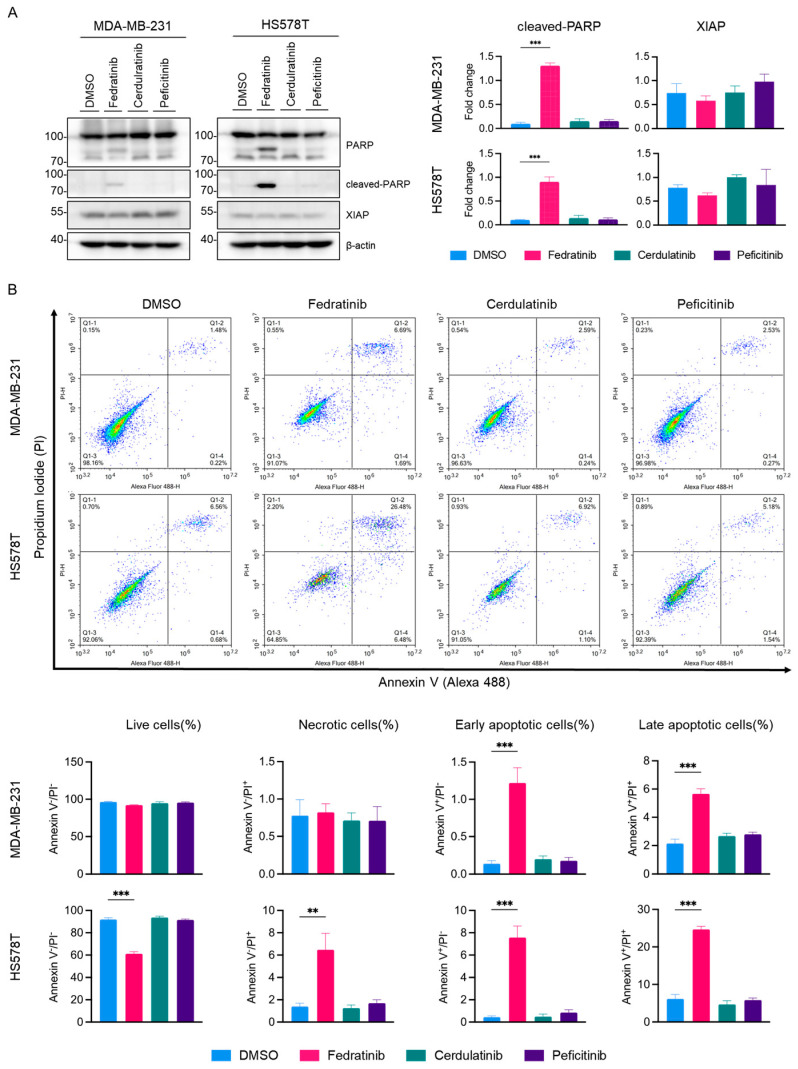
Selective induction of apoptosis by fedratinib in TNBC cells. MDA-MB-231 and HS578T were treated with 5 μM of JAK2is for 24 h. (**A**) Western blot analysis of apoptotic markers with densitometric quantification. β-actin serves as a loading control. (**B**) Fluorescence cytometric analyses using Annexin V-Alexa 488 and propidium iodide (PI) staining. Representative images, histograms, and quantification from three independent experiments are shown. Data represent mean ± SEM. ** *p* < 0.01 and *** *p* < 0.005.

To investigate whether fedratinib’s antiproliferative effect occur through apoptotic cell death, we conducted western blot analysis as shown in Figure 6. PARP cleavage was prominently detected in both MDA-MB-231 and HS578T cells treated with fedratinib, but not with cerdulatinib or peficitinib. Furthermore, fedratinib modestly reduced X-linked inhibitor of apoptosis protein (XIAP) levels in both TNBC cells lines, though this reduction did not reach statistical significance. Fluorescence cytometric analysis confirmed that only fedratinib, among tested JAK2is, induced apoptotic cell death under these conditions (Figure 6B), with marked increases in both early and late apoptotic cell populations in MDA-MB-231 and HS578T cells. Additionally, fedratinib increased the population of necrotic cells specifically in HS578T cells.

### 2.5. Inhibition of the JAK2/STAT3 Pathway Enhances the Effects of Blocking the PI3K/AKT and MEK/ERK Pathways in TNBC Cells

Based on our current and previous [25] findings, we hypothesized that while JAK2/STAT3 pathway inhibition alone may be insufficient for treatment, it could enhance the anticancer effects of the PI3K/ATK pathway, MEK/ERK pathway, or their combination in TNBC cells. Given fedratinib’s limited and cell-type-specific effects on the MEK/ERK pathway, we reasoned that more potent MEK inhibitors (MEKis) combined with JAK2is might produce enhanced therapeutic effects. To test this hypothesis, we conducted MTT cell viability assays using cerdulatinib, a JAK2i less potent than fedratinib (Figure 1), alone or in combination with either MEKi (cobimetinib [52] or trametinib [53]), and AKT inhibitor (AKTi) ipatasertib [54] or PI3K inhibitor (PI3Ki) alpelisib [55].

**Figure 7:**
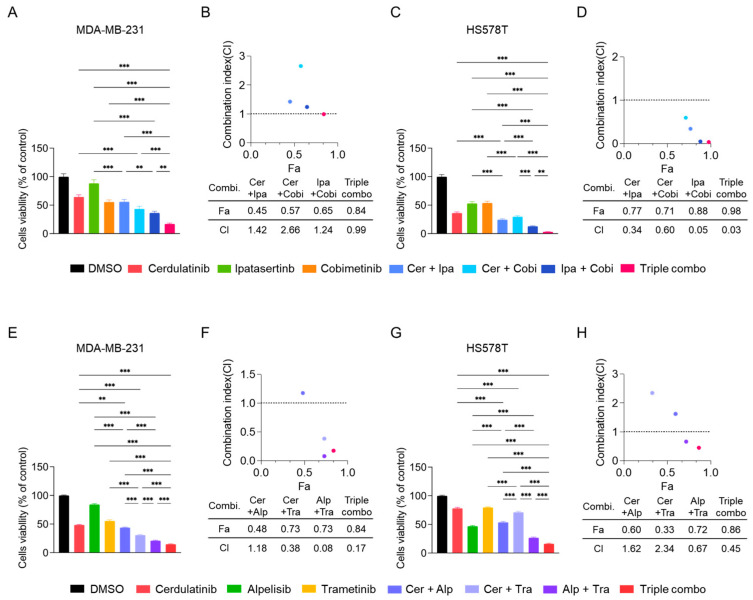
Combined inhibition of JAK, AKT/PI3K, and MEK pathways synergistically reduces TNBC cell viability. (**A**–**D**) Cell viability of TNBC cells treated with cerdulatinib (JAKi, 10 μM), ipatasertib (AKTI, 5 μM), and cobimetinib (MEKi, 5 μM) alone or in combination 72 h. (**E**,**F**) MDA-MB-231 cells were treated with cerdulatinib (5 μM), alpelisib (PI3Ki, 10 μM), and trametinib (MEKi, 10 μM) alone or in combination for 72 h. (**G**,**H**) HS578T cells were treated with cerdulatinib (10 μM), alpelisib (2.5 μM), and trametinib (10 μM) alone or in combination for 72 h. Cell viability was assessed by MTT assay. Data represent mean ± SEM from three independent experiments. ** *p* < 0.01 and *** *p* < 0.005.

In MDA-MB-231 cells, individual agents showed less potency than fedratinib (Figure 7A). The dual combination of cerdulatinib (Cer) with ipatasertib (Ipa), Cer with cobimetinib (Cobi), or Ipa with Cobi did not produce synergistic antiproliferative effects (Figure 7A,B). However, the triple combination demonstrated modest synergy with a combination index (CI) value of 0.99 (Figure 7B). HS578T cells showed more pronounced responses to combination treatments (Figure 7C,D), with dual or triple combinations achieving synergistic effects (CI values < 0.6). The triple combination exhibited particularly strong synergism in HS578T cells (Figure 7D). Similar enhanced antiproliferative effects were observed when combining cerdulatinib with alpelisib (PI3Ki) and trametinib (MEKi) (Figure 7E–H) [4]. These results indicate that the addition of JAK2i cerdulatinib further enhanced the reduction in cell viability of TNBC cells induced by combination treatment (MEKi + AKTi or MEKi + PI3Ki).

**Figure 8:**
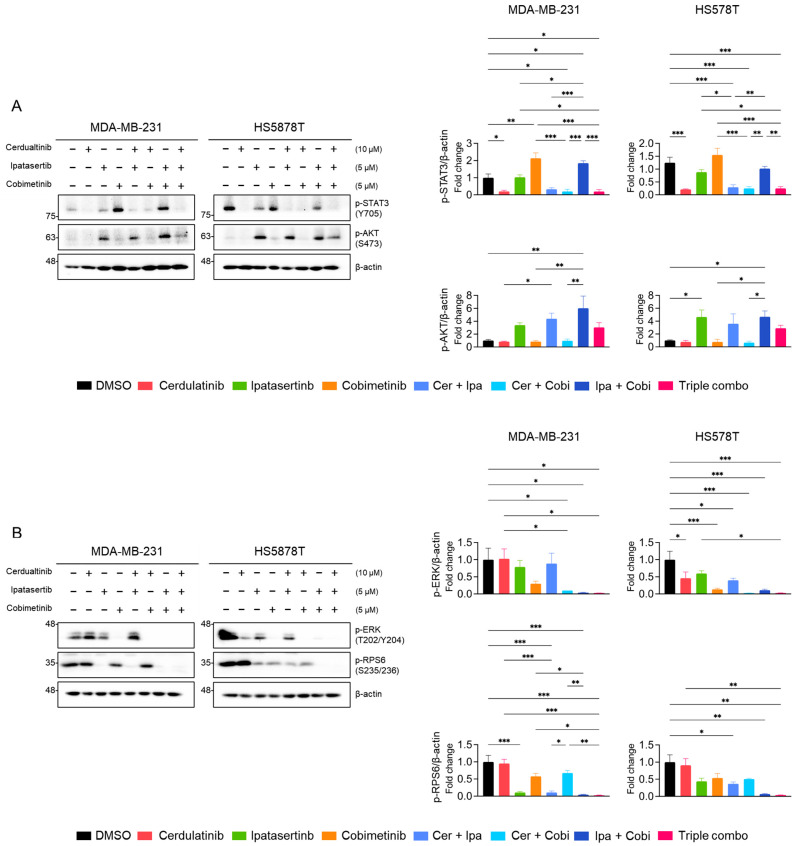
Effects of single and combined pathway inhibition on JAK2/STAT3, PI3K/AKT, and MEK/ERK signalings in TNBC cells. (**A**,**B**) Western blot analysis of signaling proteins in cells treated with the indicated PKIs for 24 h. Representative images with β-actin as a loading control and densitometric quantification from three independent experiments are shown. Data present mean ± SEM from three independent experiments. * *p* < 0.05, ** *p* < 0.01, and *** *p* < 0.005.

Western blot analyses were performed to examine the effects of combination treatments on the JAK2/STAT3, PI3K/AKT, and MEK/ERK pathways. As shown in Figure 8A, cerdulatinib consistently suppressed p-STAT3 (Y705) levels in both TNBC cell lines, while ipatasertib showed modest reduction. Interestingly, cobimetinib (MEKi) treatment induced p-STAT3 (Y705) levels in both cell lines; however, this increase was abolished by cerdulatinib co-treatment. Ipatasertib treatment elevated p-AKT (S473) levels, a previously documented effect resulting from AKT kinase inhibition [56]. This ipatasertib-induced increase in p-AKT (S473) levels remained unaffected by individual treatment with neither cerdulatinib nor cobimetinib but was reduced by combined ipatasertib and cobimetinib treatment. To further assess the PI3K/AKT pathway inhibition, we monitored p-RPS6 (S235/236) levels. Ipatasertib decreased p-RPS6 (S235/236) levels, with further reduction observed upon cobimetinib co-treatment (Figure 8B). Additionally, cobimetinib treatment completely suppressed p-ERK (T202/Y204) expression in TNBC cells (Figure 8B). These results confirmed that each selected PKIs sufficiently targets its respective pathway as intended.

**Figure 9:**
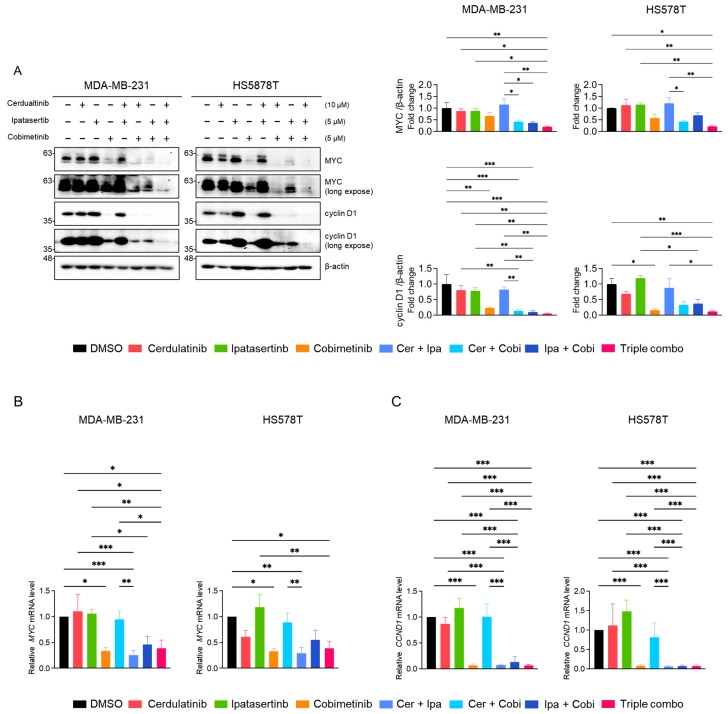
Effects of combined pathway inhibitions on MYC and cyclin D1 expression in TNBC cells. (**A**) Western blot analysis of PARP, XIAP, and caspase-3 protein levels in cells treated with indicated inhibitor combinations for 24 h, β-actin as a loading control. Representative images from three independent experiments were shown. (**B**,**C**) Relative *MYC* and *CCND1* mRNA levels measured by quantitative RT-PCR following 24 h treatment. Data represent mean ± SEM from three independent experiments. * *p* < 0.05, ** *p* < 0.01, and *** *p* < 0.005.

The effects of selected JAK2i, AKTi, MEKi, and their combinations on the expression of MYC and cyclin D1 were further evaluated using western blot and quantitative RT-PCR analysis. Among single agents, only cobimetinib modestly reduced MYC and cyclin D1 protein levels (Figure 9A). Notably, this cobimetinib-mediated protein reduction was enhanced when combined with JAK2i and AKTi. The triple combination of JAK2i, AKTi, and MEKi produced the most pronounced reduction in MYC and cyclin D1 protein levels. Unlike fedratinib, cerdulatinib did not affect the expression of mRNAs for *MYC* and *CCND1* (Figure 9B,C). Cobimetinib consistently suppressed *MYC* and *CCND1* mRNA levels in TNBC cells, though enhanced suppression through the addition of AKTi or JAK2i was observed exclusively in HS578T cells. These results indicate that JAK2 inhibition augments the suppressive effects of combined AKT and MEK inhibition on MYC and cyclin D1 primarily at the protein levels.

**Figure 10:**
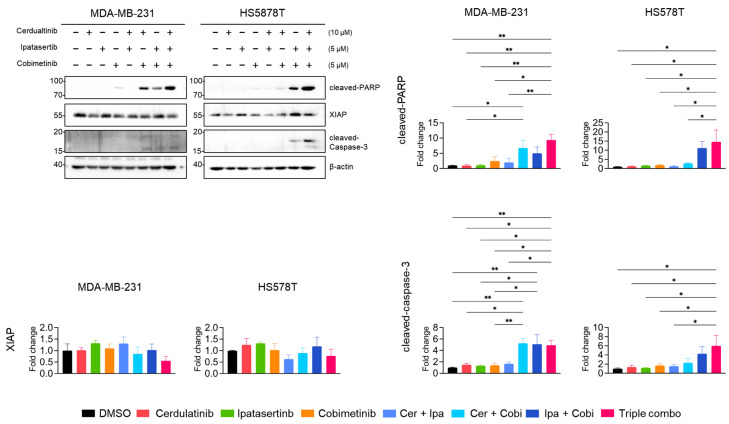
Combined pathway inhibition induced apoptotic protein expression in TNBC cells. Western blot analysis of PARP, XIAP, and caspase-3 protein levels in cells treated with indicated inhibitor combinations for 24 h, β-actin as a loading control. Representative images from three independent experiments were shown. Representative images from three independent experiments were shown. Data present mean ± SEM from three independent experiments. * *p* < 0.05 and ** *p* < 0.01.

Neither cerdulatinib nor ipatasertib triggered PARP cleavage or caspase-3 activation (Figure 10), while cobimetinib (MEKi) induced modest PARP cleavage. Notably, the dual combination of JAK2i with either MEKi (Cer + Cobi) or AKTi (Cer + Ipa) substantially enhanced PARP and caspase-3 cleavage. Consistent with MTT assay results, the tiple combination of all PKIs further augmented PARP and caspase-3 cleavage and modestly reduced XIAP levels in both MDA-MB-213 and HS578T cells. These findings suggests that while the inhibition of JAK2 alone insufficiently induces apoptotic cell death in TNBC cells, it significantly enhances the antiproliferative effects of MEKi and AKTi in TNBC cells.

## 3. Discussion

Combination chemotherapy has emerged as a widely adopted strategy for treating various cancers, aiming to achieve synergistic or additive anticancer efficacy by targeting multiple key pathways involved in cancer development [57,58,59]. For TNBC, the development of specific therapies has primarily focused on combination approach due to the molecular heterogeneity of tumor cells and intrinsic or acquired resistance of tumors to existing drugs [1,60,61,62,63]. For instance, the expression of multidrug-resistance (MDR) genes, such as ATP-binding cassette superfamily G member 2 (ABCG2), contributes to chemoresistance in TNBC [64]. Additionally, novel therapeutic entities may offer alternative approaches to conventional treatment options for TNBC [65,66,67]. In this study, we demonstrated that the JAK2/STAT3 pathway represents a potential target for enhancing the antiproliferative effects of the combined PI3K/AKT and MEK/ERK pathway inhibition.

Our analysis of TNBC cell viability using clinically approved JAKis revealed notable antiproliferative effects of fedratinib, a JAK2i. While fedratinib recently received the US FDA approval for treating myeloproliferative neoplasm-associated myelofibrosis [68], its anticancer efficacy remains largely unexplored across various human cancers, including TNBC. Given the distinct antiproliferative efficacy of fedratinib in TNBC cells compared to other JAK2is, further investigation of its effects on malignancy, metastasis, and drug resistance may provide new treatment strategies for TNBC, either as monotherapy or through synthetic lethality approaches.

Growing evidence indicates that dysregulation of the JAK/STAT pathway is implicated in multiple human cancers, including breast, gastric, lung, and ovarian cancer, hepatocellular carcinoma, and head and neck squamous cell carcinoma [69,70,71,72,73,74,75]. This pathway particularly influences tumorigenesis and chemotherapy sensitivity in TNBC [20]. Studies have shown that JAK2 genomic amplification and increased expression occur specifically in TNBC, but not ER+ and HER2+ breast tumors, correlating with poor clinical outcomes such as progression-free survival and overall survival [76]. Moreover, elevated JAK2/STAT3 pathway activation in TNBC associates with resistance to targeted therapies and promotes more invasive tumor characteristics [77,78]. The interleukin 6 (IL6)/JAK2/STAT3 pathway has also been identified as a key mediator of epithelial–mesenchymal transition (EMT) and metastasis in TNBC [79]. However, a clinical trial of ruxolitinib, a well-tolerated JAK1/2 inhibitor, in metastatic TNBC patients was discontinued due to poor clinical outcomes [80].

In this study, we demonstrated the antiproliferative effect of fedratinib on TNBC cell lines, building on our previous observation from PKI screening [4,5,6,26,27,28,29,30] (Figure 1A). Notably, while cerdulatinib and peficitinib, like fedratinib, completely abolished p-STAT3 (Y705) levels in MDA-MB-231 and HS578T cells (Figure 2A), their antiproliferative effects were less potent. These JAK2is differ in their targeting specificity: fedratinib selectively binds to the ATP-binding site of JAK2 [31]; cerdulatinib nonselectively targets JAK1, JAK2, JAK3, and TYK2 [32]; filgotinib selectively binds to the ATP-binding site of JAK1 [33]; and peficitinib acts as a pan-JAKi with moderate selectivity for JAK3 [34,35]. This comprehensive analysis of inhibitor selectivity suggests the crucial role of JAK2 in TNBC cell proliferation.

Substantial literature supports the JAK2/STAT3 pathway as a therapeutic target for TNBC [23,81]. JAK2/9p24-amplified tumors frequently occur in TNBC treated with chemotherapy, and JAK2-specific inhibitors have been reported to be more effective than dual JAK1/2 inhibitors [82]. Studies demonstrated that targeted inhibition or knockdown of the JAK2/STAT3 signaling pathway efficiently suppressed TNBC cell proliferation, migration, and invasion [83,84,85,86]. The JAK2 pathway has been implicated in the resistance to chemotherapeutic agents such as tamoxifen and paclitaxel [23,87,88]. However, our previous research showed that CP690550-mediated inhibition of STAT3 phosphorylation at Y705 alone insufficiently reduces TNBC cell proliferation [21]. CP690550, aka tofacitinib, is an approved drug which specifically target JAK3, JAK2, and JAK1 with IC_50_ value of 1, 20, and 112 nM, respectively [24,89]. In contrast, SH-I-14, a carbazole compound that inhibits p-STAT3 (Y705), demonstrated anticancer activity in TNBC cells and TNBC tumors in xenograft mouse models [90]. Further investigation revealed that CP690550 did not inhibit the proliferation of TNBC cells in MTT assays, while SH-I-14 dose-dependently reduced the TNBC cells’ proliferation through blocking STAT3 acetylation at K685 residue, disrupting the interaction between STAT3 and DNMT1, and resulting in re-repression of tumor suppressor gene expression [25]. However, since the acetylation of STAT3 (K685) is mediated by histone acetyltransferase P300 [91,92] and the mutation of K685 did not affect Y705 phosphorylation [93], we cannot directly link fedratinib to STAT3 acetylation, suggesting that phosphorylation of STAT3 Y705 may have independent roles in tumorigenesis or tumor progression.

A distinct feature of fedratinib, compared to other JAK2is used in this study, is its additionally targeting of two receptor tyrosine kinases FLT3 and RET (Table 1). This broader targeting profile aligns with our observation that only fedratinib inhibited the PI3K/AKT pathway, while cerdulatinib and peficitinib did not (Figure 2 and Figure 4). Although fedratinib also affected the MEK/ERK pathway, its efficacy showed temporal and cell-type specific limitations. Based on our previous and current findings, we hypothesized that the inhibition of the JAK2/STAT3 pathway could provide additional therapeutic benefits when combined with PI3K/AKT and/or MEK/ERK pathway inhibition. Our investigation revealed that while cerdulatinib alone minimally affected TNBC cell proliferation, its combination with MEKi or AKTi, or MEKi or PI3Ki further enhanced antiproliferative effects (Figure 7). The triple combination of JAK2i, MEKi, and AKTi or PI3Ki significantly reduced cell proliferation, markedly increased in the levels of cleaved PARP and caspase-3, and downregulated XIAP expression. This combination also reduced the expression of MYC and cyclin D1 in TNBC cells, primarily at the protein levels, warranting further investigation into the underlying molecular mechanisms.

Collectively, our findings indicate that while JAK2 inhibition alone showed limited efficacy against TNBC cell proliferation, it might potentiate the effects of combined MEK and AKT inhibition through enhanced apoptosis. However, one limitation of the current study lies in the potential adverse effects of simultaneous inhibiting these essential pathways at the systemic levels. Further preclinical and clinical validation of concurrent JAK2/STAT3, MEK/ERK, and PI3K/AKT pathway inhibition may reveal additional therapeutic advantages for treating TNBC, though careful consideration of the risk–benefit ratio will be crucial.

## 4. Materials and Methods

### 4.1. Cell Culture and Reagents

The TNBC cell lines HS578T and MDA-MB-231 were obtained from the American Type Culture Collection (ATCC; Manassas, VA, USA). Cells were cultured in Dulbecco’s modified Eagle’s medium (DMEM) supplemented with 10% heat-inactivated fetal bovine serum (FBS; Thermo Fisher Scientific, Waltham, MA, USA), 1% Antibiotic-Antimycotic (Thermo Fisher Scientific), and 5 μg/mL anti-mycoplasma (GenDEPTO, Baker, TX, USA) at 37 °C in a humidified atmosphere containing 5% CO_2_. Cell viability and counting were assessed using the trypan blue (Sigma-Aldrich, St. Louis, MO, USA) exclusion method and a LUNA II Automated Cell Counter (Logos Biosystems, Anyang-si, Gyeonggi-Do, Korea) as described previously [94]. JAK2is (fedratinib, ceduratinib, peficitinib, and filgotinib), MEKis (cobimetinib and trametinib), an AKTi (ipatasertib), and a PI3Ki (alpelisib) were purchased from Selleck Chemicals (Houston, TX, USA). Compounds were dissolved in dimethyl sulfoxide (DMSO) (Duchefa Biochemie, Haarlem, The Netherlands) and stored at −20 °C in small aliquots.

### 4.2. MTT Assay

For cell viability assays, TNBC cells were seeded at 2 × 10^3^ cells per well in 96-well plates and allowed to adhere overnight before treatment. Cells were treated with increasing concentrations of the indicated compounds for 72 h. MTT reagent (3-(4,5-Dimethylthiazol-2-yl)-2,5-Diphenyltetrazolium Bromide) was added to a final concentration of 0.5 mg/mL and viable cells to measure cell viability as previously described [26,95,96]. The cells were incubated at 37 °C for 4 h, culture medium was removed, and the formazan crystals were dissolved in DMSO. Absorbance was measured at 560 nm using an iMARK Microplate Absorbance Reader (Bio-Rad, Hercules, CA, USA). All experiments were performed in triplicate and independently repeated at least three times.

### 4.3. Clonogenic Survival Assay

For colony formation assays, TNBC cells were at 1 × 10^3^ cells per well in 6-well plates. At the next day, cells were treated with increasing concentrations of fedratinib or 5 μM of fedratinib, cerdulatinib, or peficitinib for 24 h. Following treatment, cells were washed and cultured in fresh normal growth medium for an additional 10–14 days to allow colony formation. Colonies were fixed and stained with crystal violet solution, and then washed and air dried at room temperature. Stained colonies were imaged with a flatbed scanner. For quantification, crystal violet was solubilized in a 1:1 mixture (*v*/*v*) of 0.1 M sodium phosphate buffer (pH4.5) and ethanol, and absorbance was measure as previously described [4,5,6].

### 4.4. Western Blot Analysis

Western blot analyses were performed as previously described [30]. In brief, TNBC cells were treated with the indicated compounds under specified conditions, harvested, and lysed on ice using RIPA buffer supplemented with protease and phosphatase inhibitors (ThermoFisher Scientific). Protein concentrations were determined using the bicinchoninic acid (BCA) protein assay (Thermo Fisher Scientific). Equal amounts of proteins were separated by SDS-PAGE and transferred to a polyvinylidene difluoride (PVDF) membrane (Sigma-Aldrich). The following antibodies were used in this study (Table 2).

### 4.5. Fluorescence Cell Flow Cytometry

MDA-MB-231 and HS578T cells (5 × 10^5^ cells per 60 mm dish) were seeded and incubated at 37 °C in a humidified atmosphere containing 5% CO_2_ for 24 h. Subsequently, the medium was replaced with fresh medium containing 5 µM of fedratinib, cerdulatinib and peficitinib, and cells were incubated for an additional 24 h. Following treatment, cells were harvested using TrypLE™ Express (Thermo Fisher Scientific), washed twice with cold Dulbecco’s phosphate buffered saline (DPBS), and centrifuged at 2000 rpm for 5 min. Cell pellets were resuspended in 100 µL of 1× Annexin V buffer (Cell Signaling Technology), 5 µL of Annexin V-Alexa 488 (Thermo Fisher Scientific), and 12.5 µL of PI (Cell Signaling Technology) were added to each sample, followed by incubation on ice in the dark for 15 min. Apoptotic and necrotic cells were quantified using a BD FACS Calibur™(Becton Dickinson sciences, Franklin Lakes, NJ, USA) system and CellQuest™ Pro software (BD, version 4.0.2) or NovoCyte Flow Cytometer System (Agilent Technologies, Inc., Santa Clara, CA, USA).

### 4.6. RNA Extraction, cDNA Synthesis, and Quantitative Real-Time PCR (RT-PCR)

Total RNA was extracted from cultured TNBC cells using the RNeasy Mini Kit (Cat. No. 74124, Qiagen, Hilden, Germany) according to the manufacturer’s protocol. RNA concentration and purity were measured using a BioSpectrometer (Eppendorf, Hamburg, Germany). For cDNA synthesis, 1 μg of total RNA was reverse-transcribed in a 20 μL reaction volume using the ReverTra Ace™ qPCR RT Kit & Master Mix (Cat. No. FSQ-101, TOYOBO, Osaka, Japan), containing 250 ng of random primers. The synthesized cDNA was diluted 1:50 with nuclease-free water and stored at –20 °C until further use.

Quantitative RT-qPCR was performed using the iTaq™ Universal SYBR^®^ Green Supermix (Cat. No. 1725121, Bio-Rad Laboratories, Hercules, CA, USA) on an Applied Biosystems StepOne™ Real-Time PCR System (Thermo Fisher Scientific). Each 10 μL PCR reaction mixture consisted of 5 μL SYBR Green Supermix, 1 μL diluted cDNA, 0.4 μM of each gene-specific forward and reverse primer, and nuclease-free water to a final volume of 10 μL. The thermal cycling conditions were as follows: initial denaturation at 95 °C for 30 s, followed by 40 cycles of 95 °C for 5 s and 60 °C for 30 s. A melting curve analysis was included to confirm the specificity of the PCR products. The following primers were used in this study: CCND1 (Cyclin D1, Gene ID: 595), forward: 5′-AGCTCCTGTGCTGCGAAGTGGAAAC-3′ and reverse: 5′-AGTGTTCAATGAAATCGTGCGGGGT-3′; MYC (Gene ID: 4609), forward: 5′-GGCTCCTGGCAAAAGGTCA-3′ and reverse: 5′-CTGCGTAGTTGTGCTGATGT-3′; and ACTB (β-actin, internal control), forward: 5′-CACCATTGGCAATGAGCGGTTC-3′ and reverse: 5′-AGGTCTTTGCGGATCTCCACGT-3′. Relative gene expression was calculated using the 2^–ΔΔCt method with ACTB as the reference gene. All qPCR reactions were performed in triplicate for each sample to ensure reproducibility.

### 4.7. Statistical Analysis

All experiments were performed at least thrice. Data are presented as mean ± SEM. The Shapiro–Wilk test was used to check the suitability of parametric methods. After confirming, one-way repeated measures analysis of variance (ANOVA) was conducted to calculate *p*-values. Dunnett’s test was used as a post hoc test to compare data between groups. All statistical analyses were performed using GraphPad Prism Ver 10.3.1 (GraphPad Software, Boston, MA, USA). Statistically significant differences are indicated as follows: * *p* < 0.05, ** *p* < 0.01, and *** *p* < 0.005.

## Figures and Tables

**Table 1:** Information on the clinically approved JAK2is used in this study.

PKI (Ref)	Known Targets (IC_50_ Values in nM)		IC50 in HS578T (μM)	IC50 in MDA-MB-231 (μM)
	JAK Family	Other PKs		
Fedratinib [31]	JAK2 (3), JAK1 (105 *), JAK3 (1002 *)	FLT3 (15), RET (48)	1.23 ± 0.19	1.38 ± 0.06
Cerdulatinib [32]	JAK1 (12), JAK2 (6), JAK3 (8), TYK2 (0.5)	SYK (32)	9.66 ± 1.15	8.07 ± 0.62
Filgotinib [33]	JAK1 (10), JAK2 (28), JAK3 (810), TYK2 (116)		>10	>10
Peficitinib [34,35]	JAK1 (3.9), JAK2 (5.0), JAK3 (0.71), TYK2 (4.8)		>10	8.75 ± 0.58

* calculated by multiplying IC_50_ value of JAK2 by fold selectivity of each JAK family member [31].

**Table 2:** Antibodies used in this study.

Name	Cat #	M.W. (kDa)	Source	Company
AKT	9272	60	rabbit	Cell Signaling Technology (Denver, MA, USA)
p-AKT (S473)	9271	60	rabbit	
ERK1/2	9102	42, 44	rabbit	
p-ERK1/2 (T202/Y204)	9101	42, 44	rabbit	
mTOR (L27D4)	4517	289	mouse	
p-mTOR (S2448)	5536	289	rabbit	
RPS6	2217	32	rabbit	
p-RPS6 (S235/236)	4856	32	rabbit	
STAT3	9139	78, 86	mouse	
p-STAT3 (Y705)	9145	79, 86	rabbit	
4E-BP1	9452	15, 20	rabbit	
p-4E-BP1 (T37/46)	2855	15, 20	rabbit	
PRAS40	2691	40	rabbit	
p-PRAS40 (T246)	13175	40	rabbit	
XIAP	2042	53	rabbit	
PARP	9542	89, 116	rabbit	
MYC	5605	62	rabbit	
Cyclin D1	55506	36	Rabbit	
Cleaved PARP	5625	89	rabbit	
Cleaved caspase-3	9664	17, 19	rabbit	
β-actin	SC-47778	43	mouse	Santa Cruz Biotechnology (Dallas, TX, USA)

## Data Availability

The data and materials in the present study may be available from the corresponding author upon reasonable requests.

## Supplementary Materials

The following supporting information can be downloaded at: https://www.mdpi.com/article/10.3390/ijms26136139/s1.

## Abbreviations

The following abbreviations are used in this manuscript:

ADC	antibody drug conjugate
AKTi	AKT inhibitor
ANOVA	analysis of variance
ATCC	American Type Culture Collection
DMEM	Dulbecco’s modified Eagle’s medium
DMSO	dimethyl sulfoxide
DNMT1	DNA (cytosine-5)-methyltransferase 1
DPBS	Dulbecco’s phosphate buffered saline
EGFR	epidermal growth factor receptor
ER	estrogen receptor
ERK	extracellular signal-regulated kinase
FBS	fetal bovine serum
FDA	Food and Drug Administration
FLT3	FMS-like tyrosine kinase 3
GvHD	graft-versus-host disease
HER2	human epidermal growth factor 2
IL6	interleukin 6
JAK	Janus kinase
JAK2i	JAK2 inhibitor
MEK	MAP kinase/ERK kinase
MEKi	MEK inhibitor
mTOR	mammalian target of rapamycin
MTT	3-(4,5-Dimethylthiazol-2-yl)-2,5-Diphenyltetrazolium Bromide
PARP1	poly [adenosine diphosphate (ADP)-ribose] polymerase 1
PD-L1	programmed cell death ligand 1
PDLIM4	PDZ and LIM domain protein 4
PKI	protein kinase inhibitor
PR	progesterone receptor
PVDF	polyvinylidene difluoride
RET	rearranged during transfection
RTK	receptor tyrosine kinase
STAT3	signal transducer and activator of transcription 3
SYK	spleen tyrosine kinase
TNBC	triple-negative breast cancer
TYK2	tyrosine kinase 2
VHL	von Hippel-Lindau disease tumor suppressor
XIAP	X-linked inhibitor of apoptosis protein
