# Supplementary material for: JAK2 Inhibition Augments the Anti-Proliferation Effects by AKT and MEK Inhibition in Triple-Negative Breast Cancer Cells
Source: Int J Mol Sci. 2025 Jun 26;26(13):6139. doi: 10.3390/ijms26136139 (PMC12250121; doi:10.3390/ijms26136139)
Supplement: Supplementary file 1 [file ijms-26-06139-s001.zip › ijms-3487647-supplementary.pdf]

JAK2 inhibition augments the anti-proliferation effects by AKT and MEK inhibition in triple-negative breast cancer cells

Kyu Sic You [1,2,*], Tae-Sung Kim [1,2,*], Su Min Back [1], Jeong-Soo Park [1,2], Kangdong Liu [3,4], Yeon-Sun Seong [1,2], Dong Joon Kim [2,3,4,5,#] and Yong Weon Yi [1,2,#]

Original Blot Data Used in the Manuscript

Example: Blot Presentation for Manuscript

Final Figures

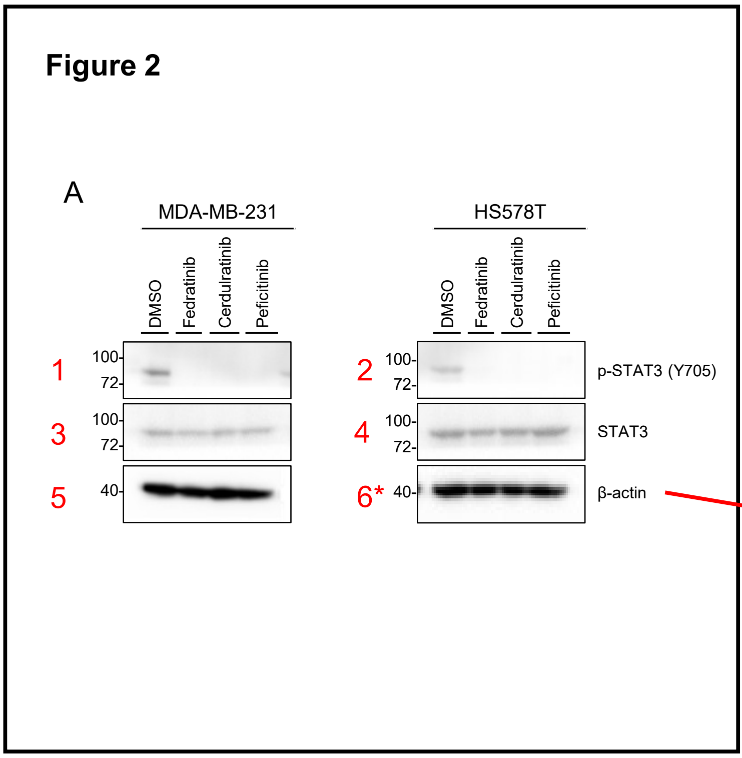

*Blot Identity (Red Numbers)

Original Uncropped Blot Images

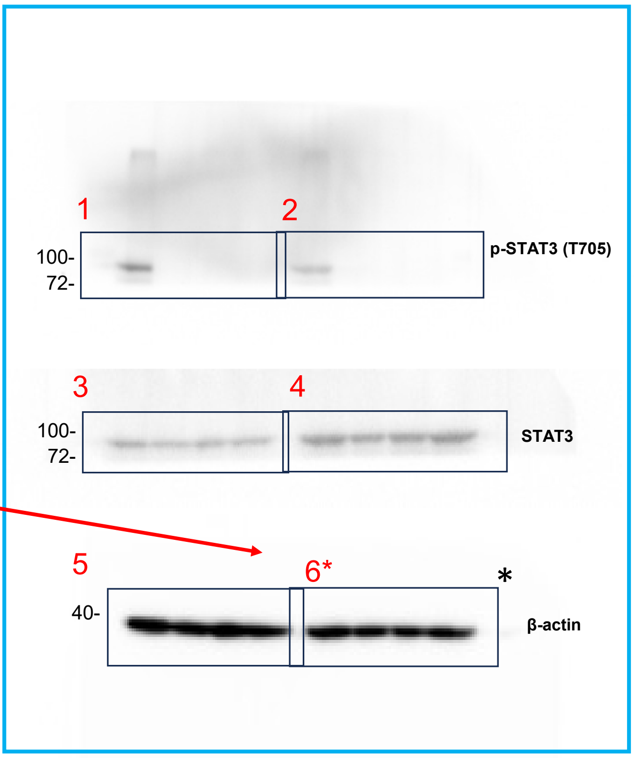

*Cropped panels used in the final figure

Figure S2

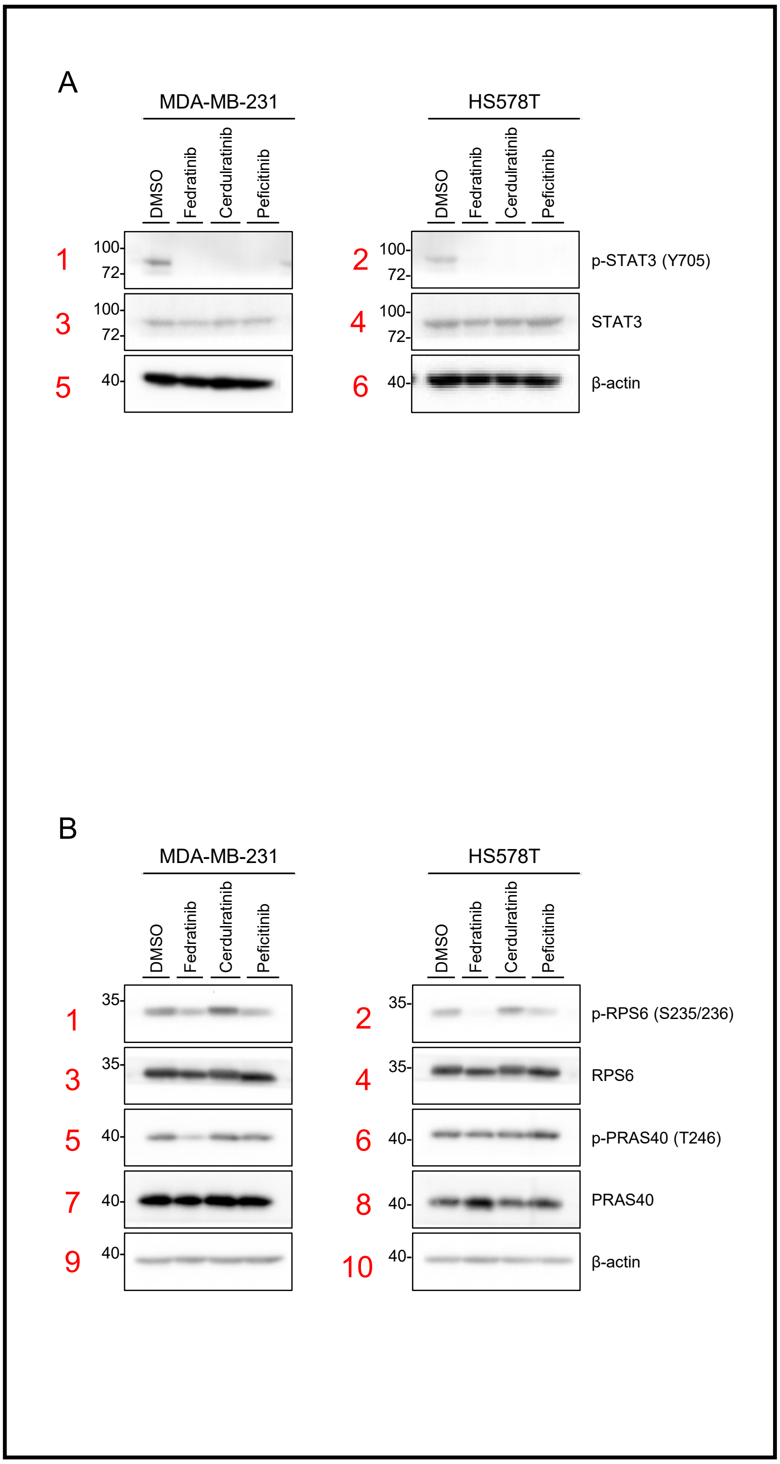
A
MDA-MB-231
HS578T
DMSO
Fedratinib
Cerdulratinib
Peficitinib
100-
72-
40-
1
2
3
4
5
6
p-STAT3 (Y705)
STAT3
β-actin
B
MDA-MB-231
HS578T
DMSO
Fedratinib
Cerdulratinib
Peficitinib
35-
40-
1
2
3
4
5
6
7
8
9
10
p-RPS6 (S235/236)
RPS6
p-PRAS40 (T246)
PRAS40
β-actin

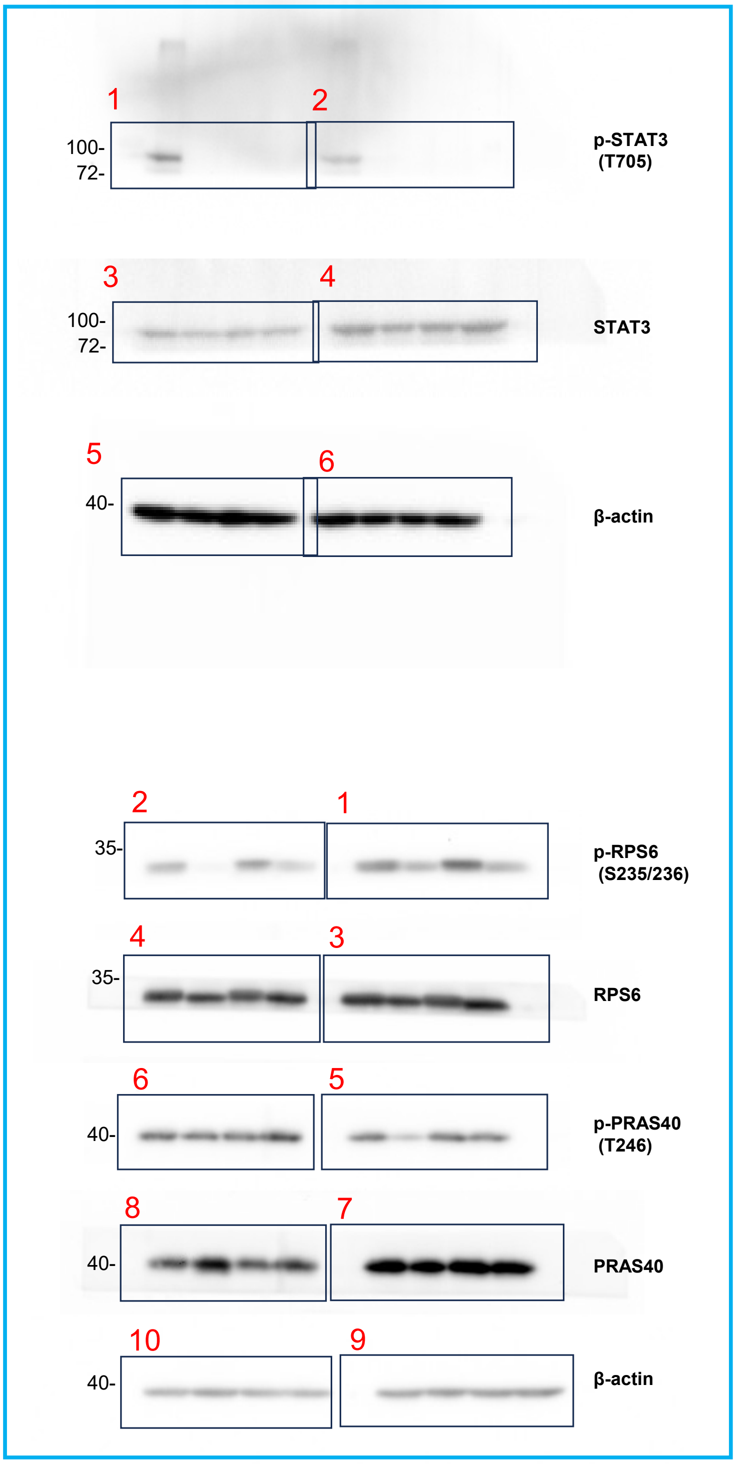
1
2
100-
72-
p-STAT3 (T705)
3
4
100-
72-
STAT3
5
6
40-
β-actin
2
1
35-
p-RPS6 (S235/236)
4
3
35-
RPS6
6
5
40-
p-PRAS40 (T246)
8
7
40-
PRAS40
10
9
40-
β-actin

Figure S3

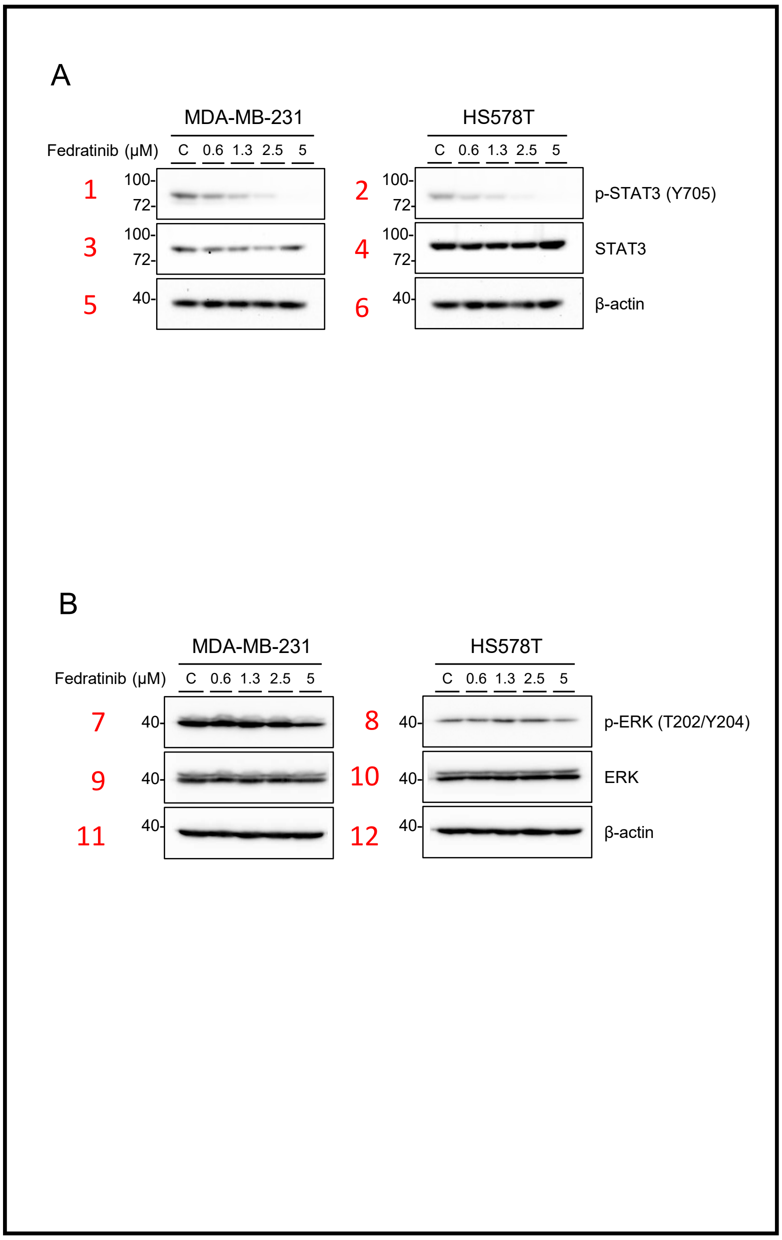
A
MDA-MB-231
HS578T
Fedratinib (µM) C 0.6 1.3 2.5 5
p-STAT3 (Y705)
STAT3
β-actin
B
MDA-MB-231
HS578T
Fedratinib (µM) C 0.6 1.3 2.5 5
p-ERK (T202/Y204)
ERK
β-actin

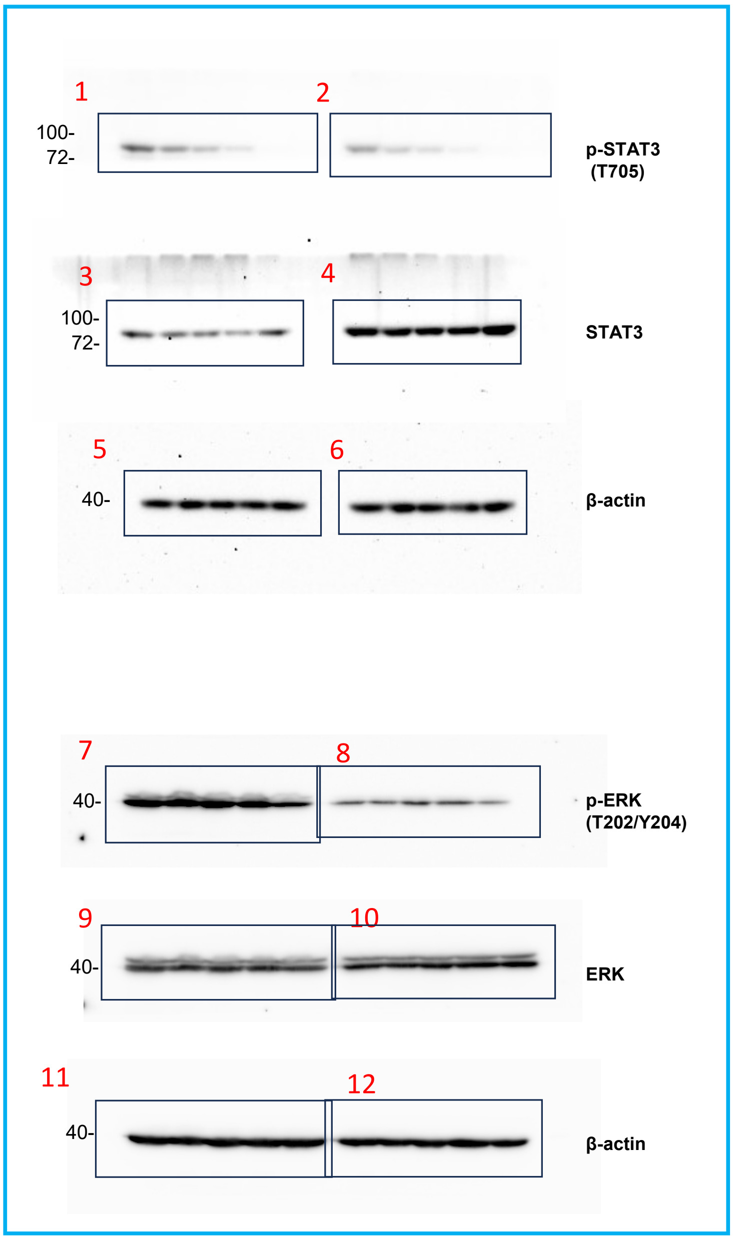
p-STAT3 (T705)
STAT3
β-actin
p-ERK (T202/Y204)
ERK
β-actin

Figure S3

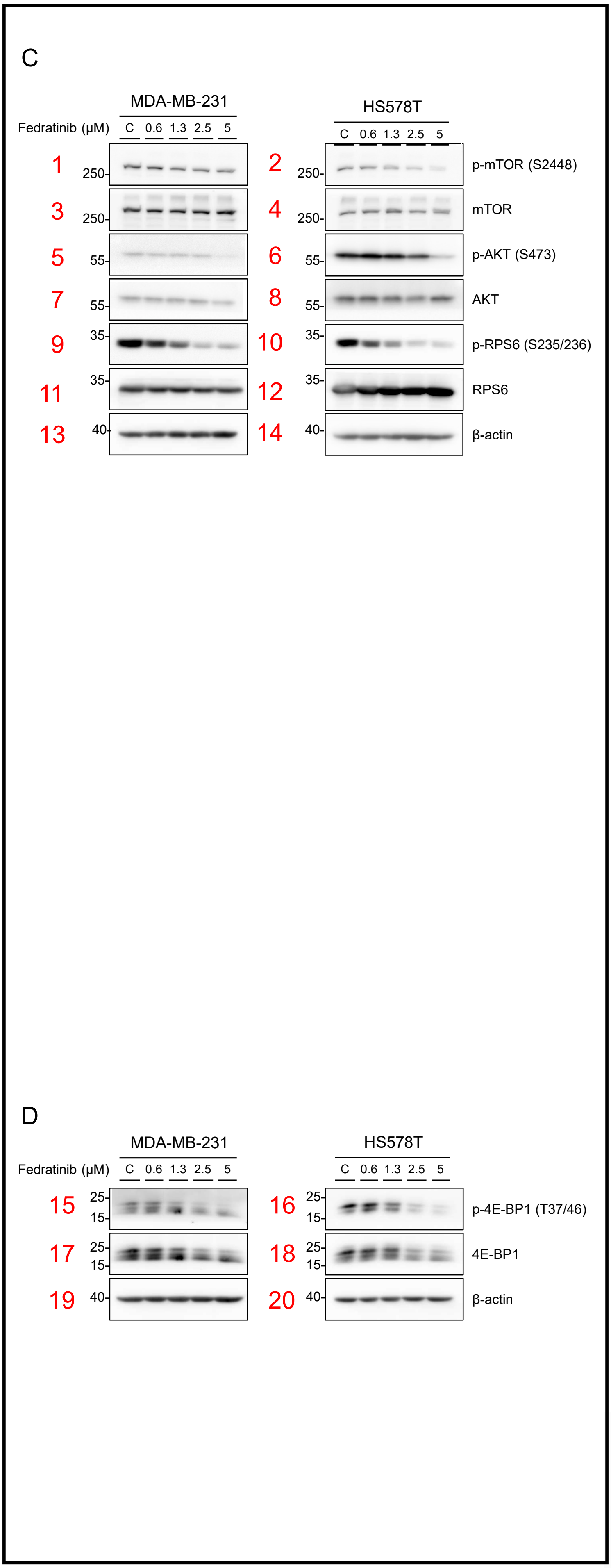
C
MDA-MB-231
HS578T
Fedratinib (μM)
C 0.6 1.3 2.5 5
p-mTOR (S2448)
mTOR
p-AKT (S473)
AKT
p-RPS6 (S235/236)
RPS6
β-actin
D
MDA-MB-231
HS578T
Fedratinib (μM)
C 0.6 1.3 2.5 5
p-4E-BP1 (T37/46)
4E-BP1
β-actin

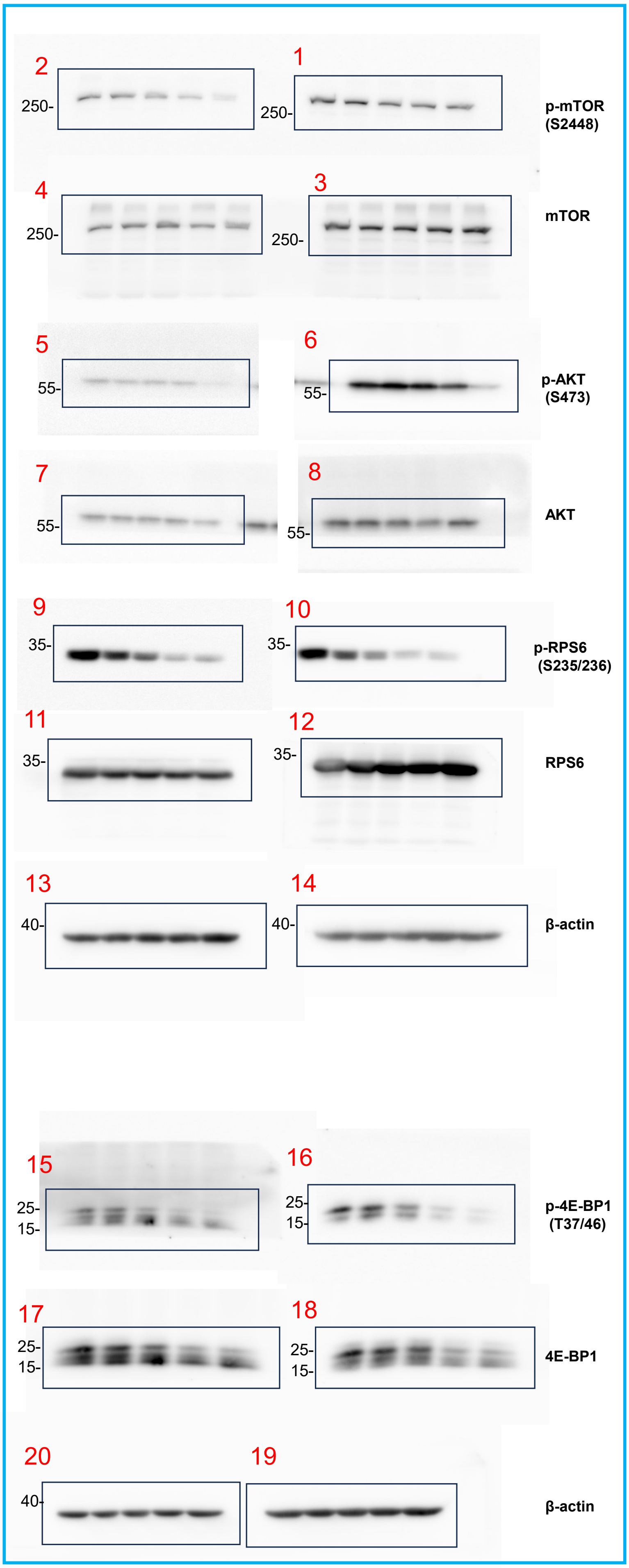
p-mTOR (S2448)
mTOR
p-AKT (S473)
AKT
p-RPS6 (S235/236)
RPS6
β-actin
p-4E-BP1 (T37/46)
4E-BP1
β-actin

Figure S4

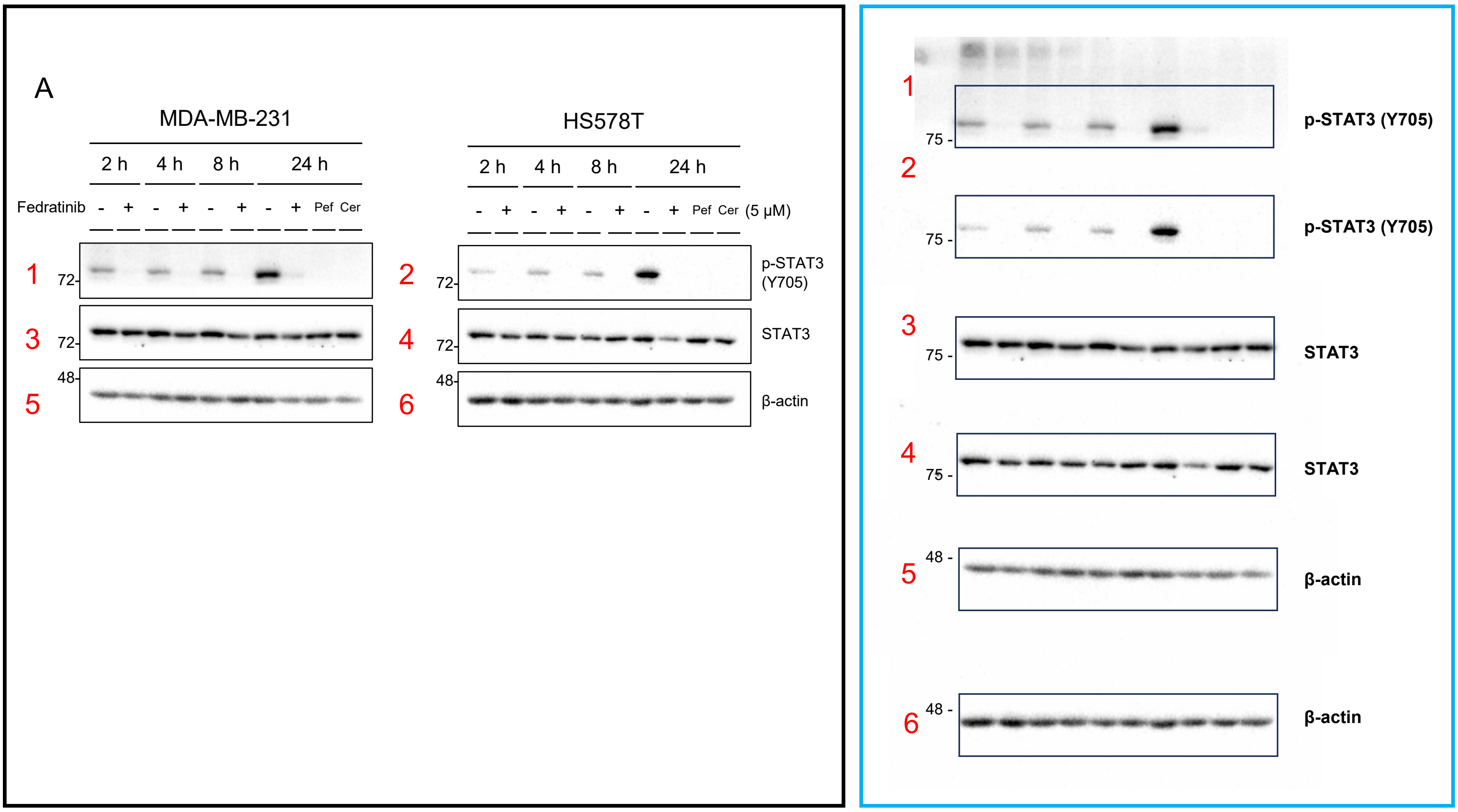
A
MDA-MB-231
2 h
4 h
8 h
24 h
Fedratinib
-
+
Pef
Cer
HS578T
(5 μM)
72-
48-
p-STAT3 (Y705)
STAT3
β-actin
1
2
3
4
5
6
75 -
48 -
p-STAT3 (Y705)
STAT3
β-actin

Figure S4

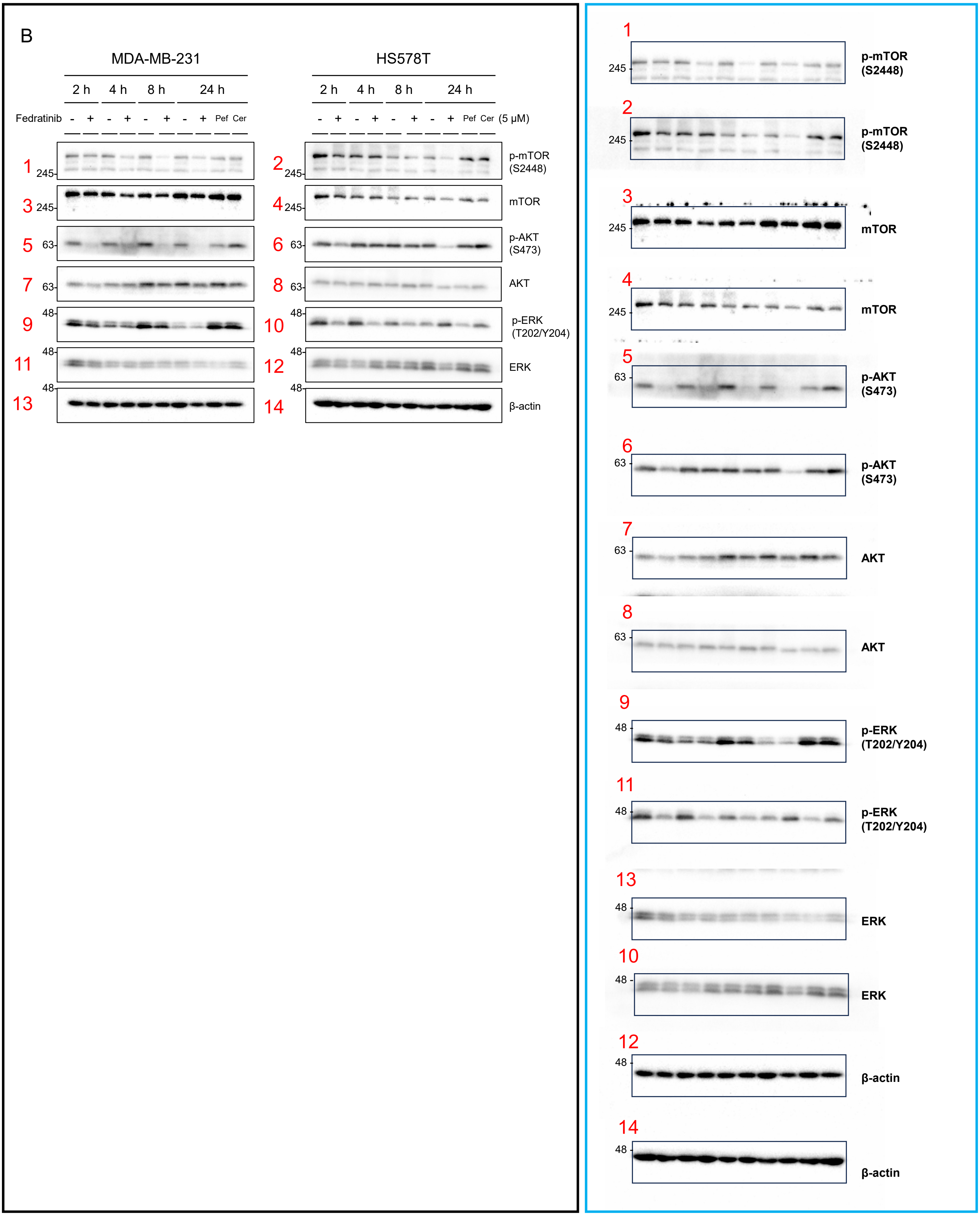
B
MDA-MB-231
HS578T
2 h
4 h
8 h
24 h
Fedratinib
Pef
Cer
(5 μM)
245
63
48
p-mTOR (S2448)
mTOR
p-AKT (S473)
AKT
p-ERK (T202/Y204)
ERK
β-actin
1
2
3
4
5
6
7
8
9
10
11
12
13
14

Figure S5

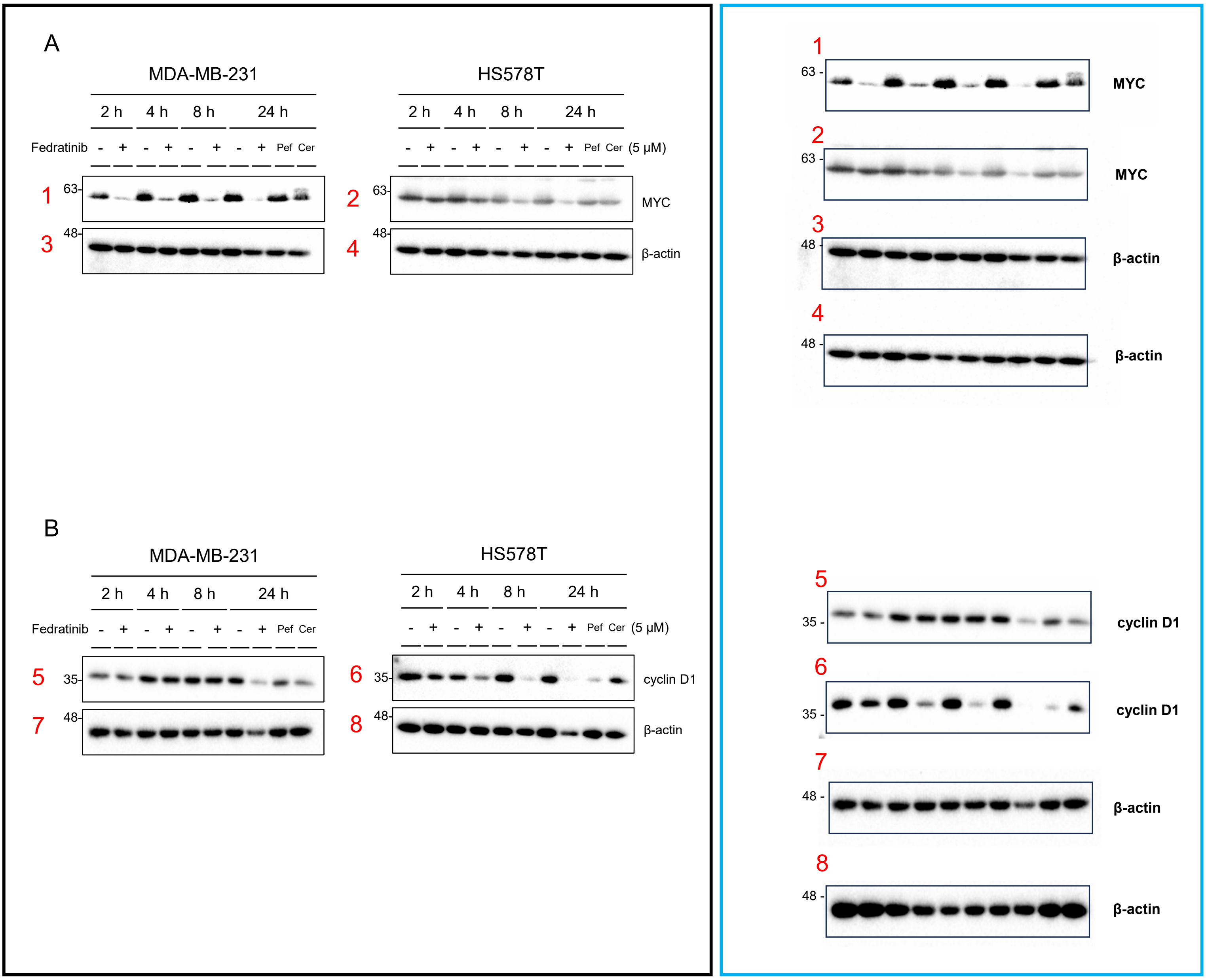
A
MDA-MB-231
HS578T
2 h
4 h
8 h
24 h
Fedratinib
Pef
Cer
(5 μM)
63-
48-
MYC
β-actin
B
35-
cyclin D1
1
2
3
4
5
6
7
8

Figure S6

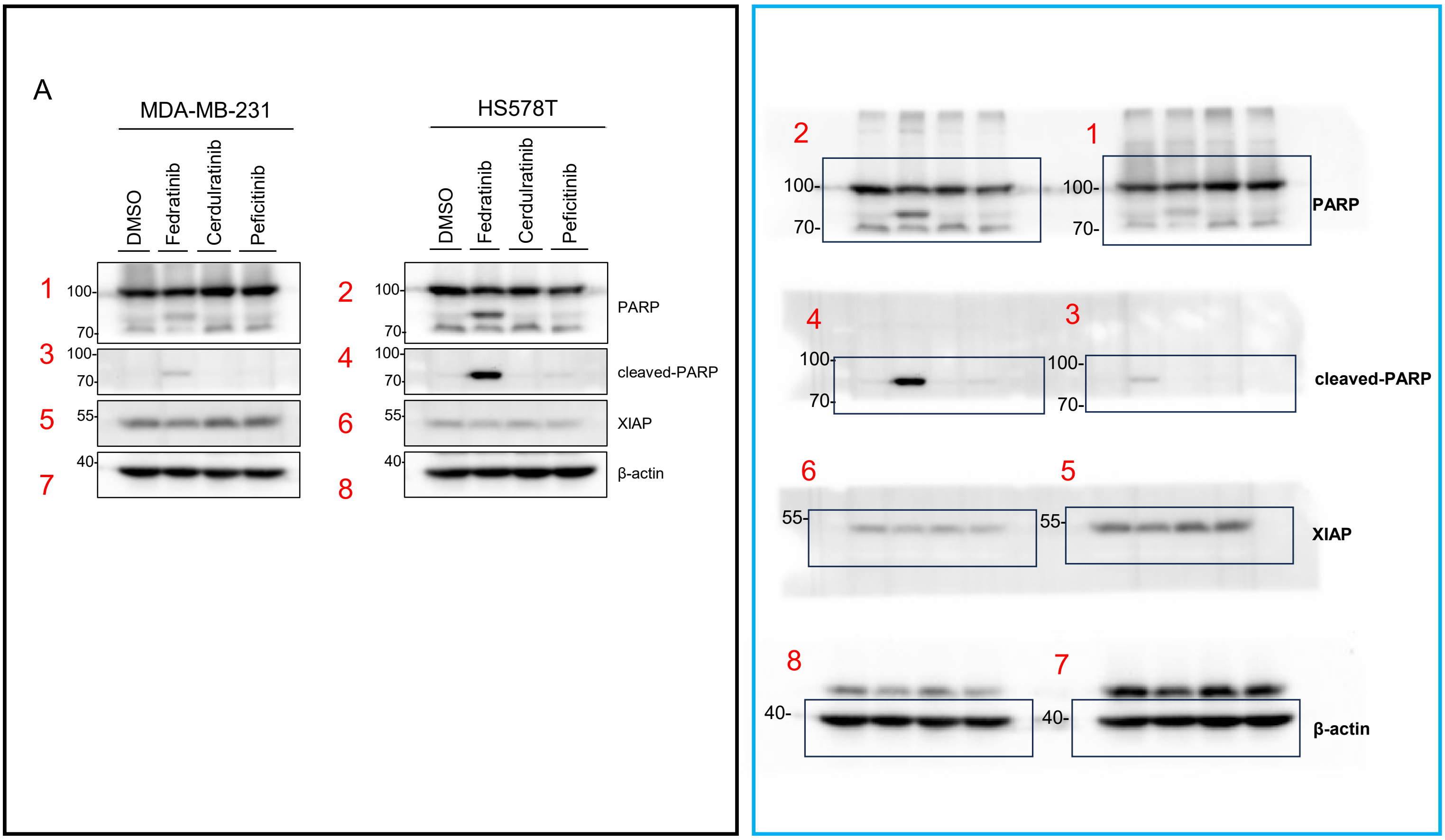
A
MDA-MB-231
HS578T
DMSO
Fedratinib
Cerdulratinib
Peficitinib
PARP
cleaved-PARP
XIAP
β-actin
100-
70-
55-
40-

Figure S8

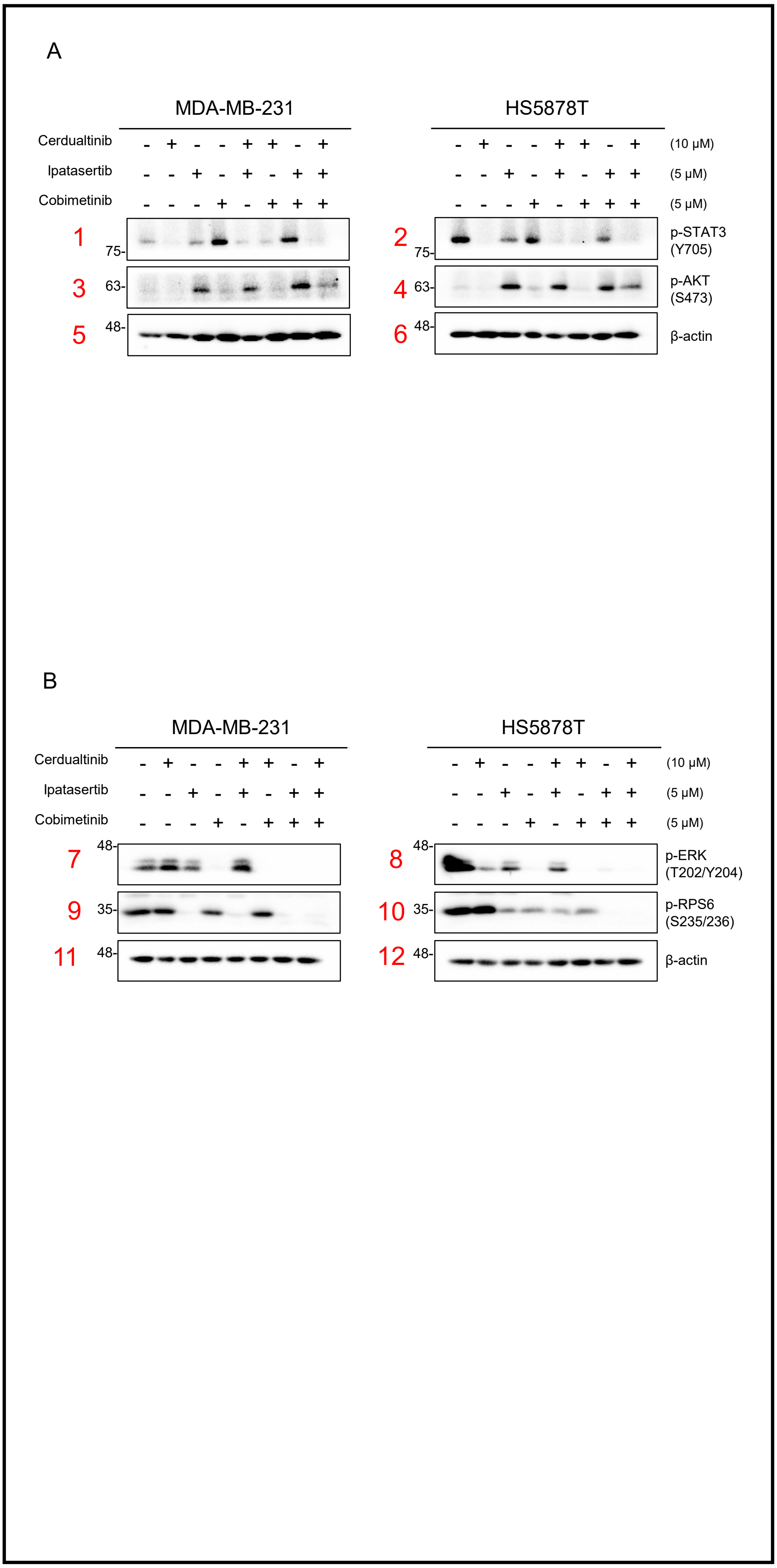

A
MDA-MB-231
HS5878T
Cerdualtinib
Ipatasertib
Cobimetinib
(10 μM)
(5 μM)
(5 μM)
p-STAT3 (Y705)
p-AKT (S473)
β-actin
B
MDA-MB-231
HS5878T
Cerdualtinib
Ipatasertib
Cobimetinib
p-ERK (T202/Y204)
p-RPS6 (S235/236)
β-actin

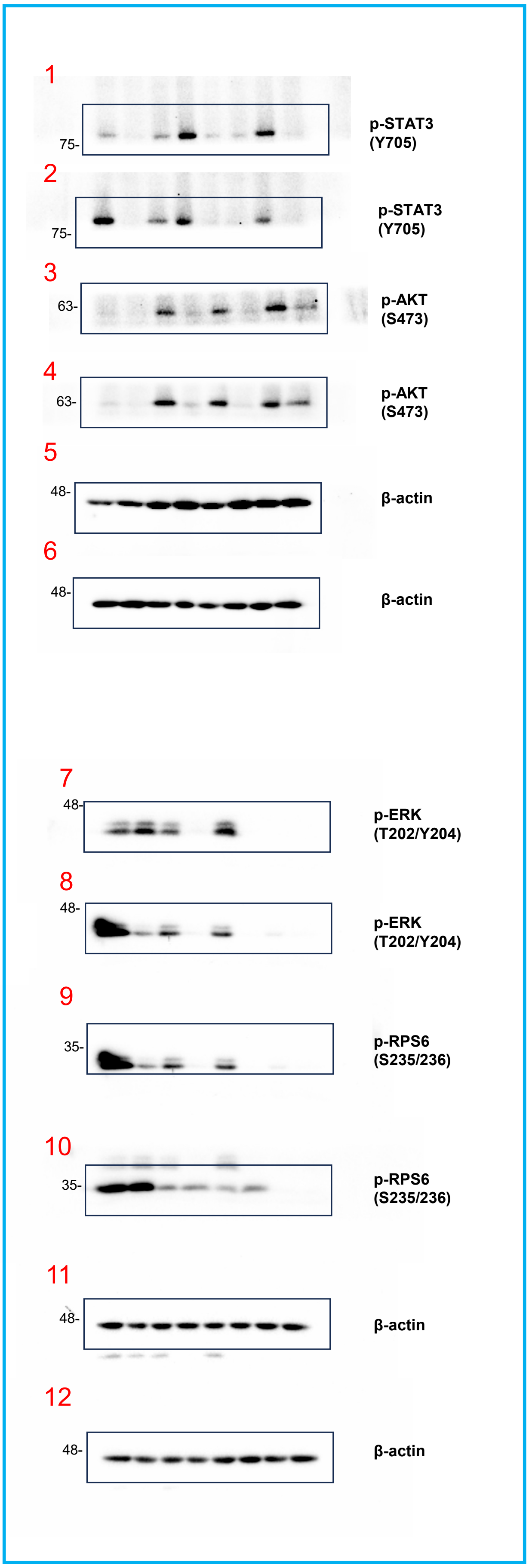

p-STAT3 (Y705)
p-STAT3 (Y705)
p-AKT (S473)
p-AKT (S473)
β-actin
β-actin
p-ERK (T202/Y204)
p-ERK (T202/Y204)
p-RPS6 (S235/236)
p-RPS6 (S235/236)
β-actin
β-actin

Figure S9

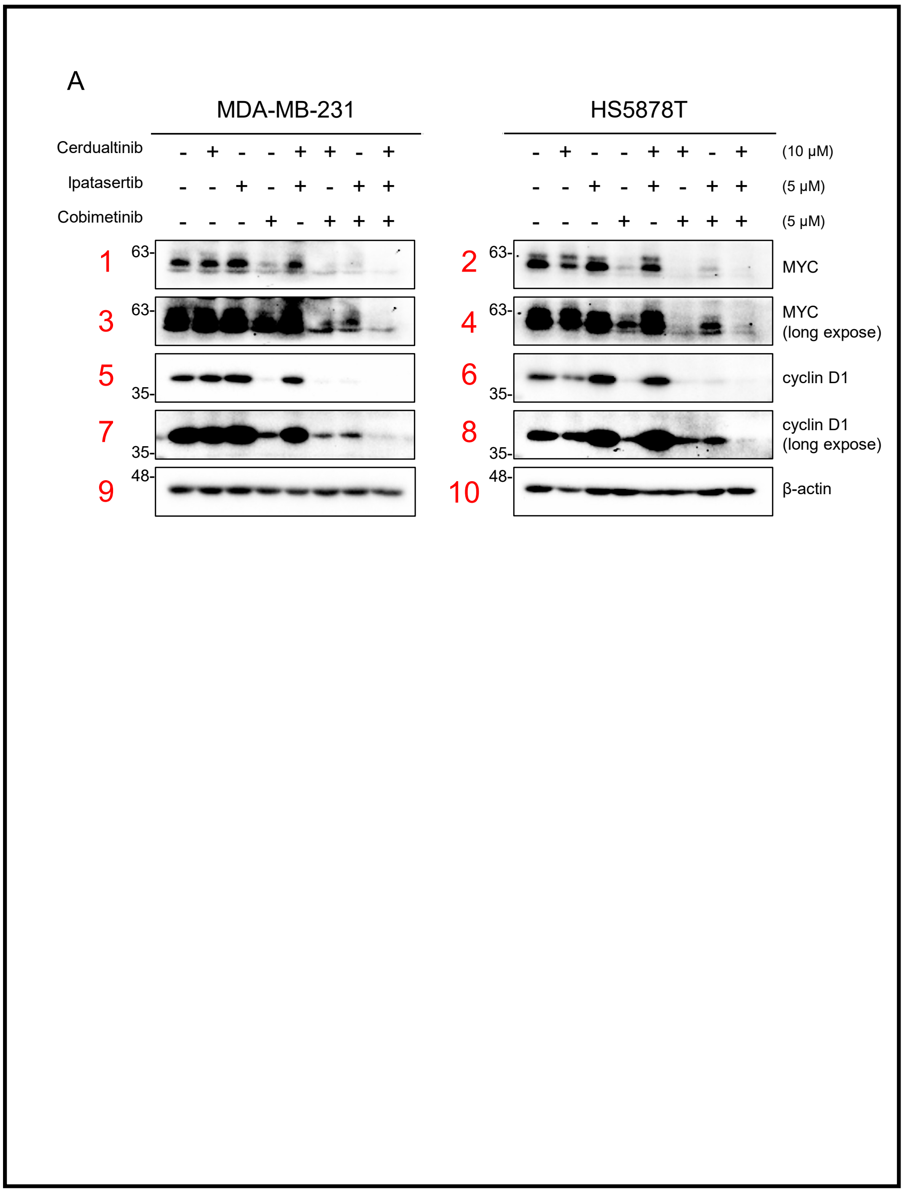

A
MDA-MB-231
HS5878T
Cerdualtinib
Ipatasertib
Cobimetinib
(10 μM)
(5 μM)
(5 μM)
MYC
MYC (long expose)
cyclin D1
cyclin D1 (long expose)
β-actin

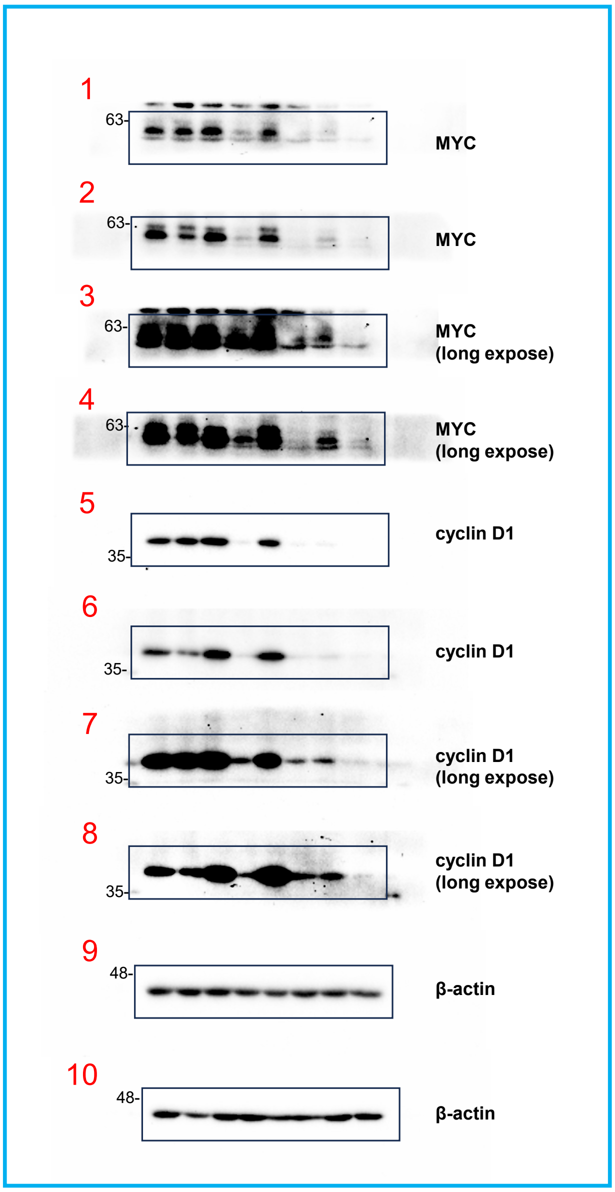

MYC
MYC (long expose)
cyclin D1
cyclin D1 (long expose)
β-actin

Figure S10

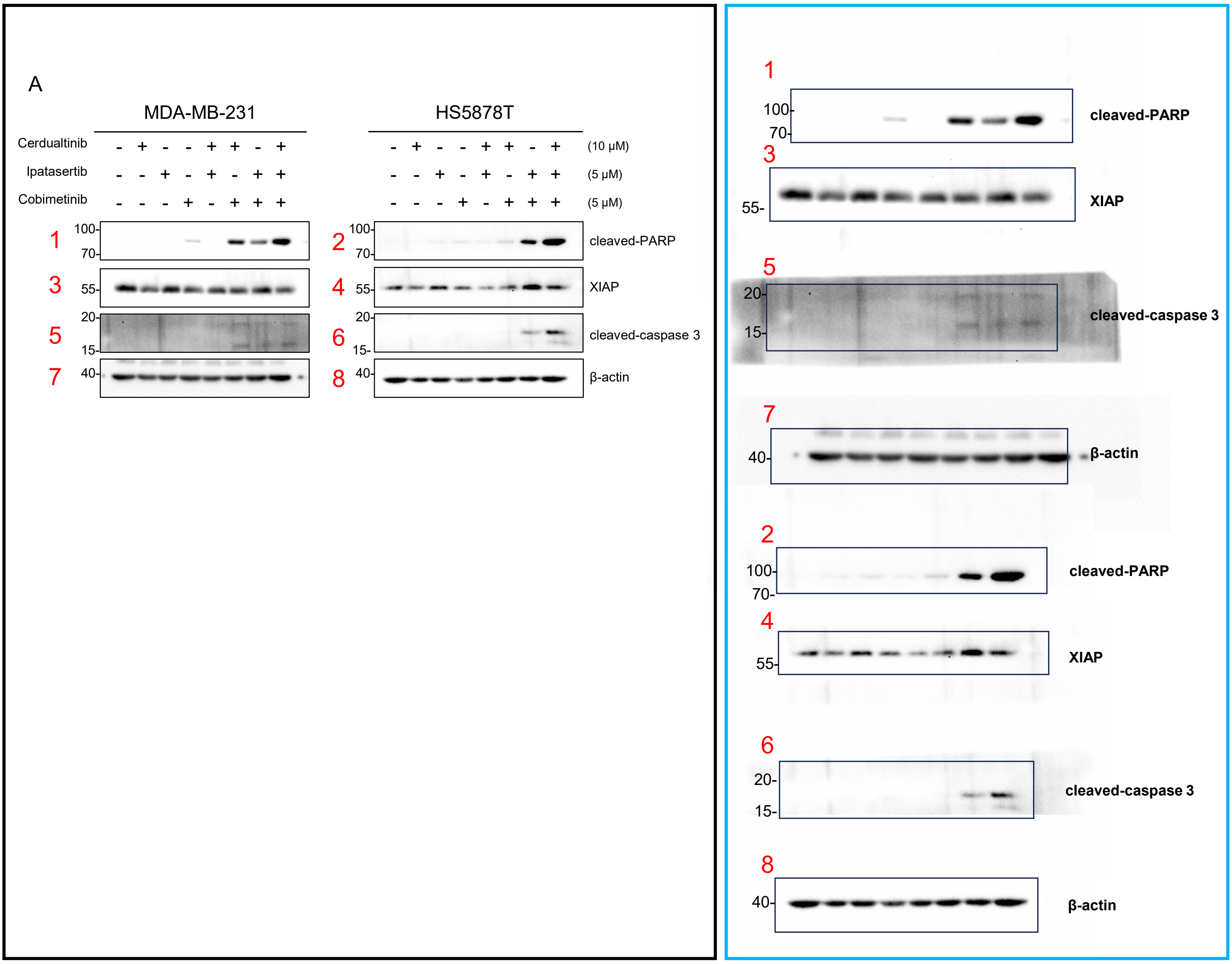
A
MDA-MB-231
HS5878T
Cerdualtinib
Ipatasertib
Cobimetinib
(10 μM)
(5 μM)
(5 μM)
cleaved-PARP
XIAP
cleaved-caspase 3
β-actin
100-
70-
55-
20-
15-
40-
1
2
3
4
5
6
7
8
